# Comprehensive investigation of p53, p21, nm23, and VEGF expression in hepatitis B virus-related hepatocellular carcinoma overall survival after hepatectomy

**DOI:** 10.7150/jca.33766

**Published:** 2020-01-01

**Authors:** Guang-Zhi Zhu, Xi-Wen Liao, Xiang-Kun Wang, Yi-Zhen Gong, Xiao-Guang Liu, Long Yu, Chuang-Ye Han, Cheng-Kun Yang, Hao Su, Ke-Tuan Huang, Ting-Dong Yu, Jian-Lu Huang, Jia Li, Zhi-Ming Zeng, Wei Qin, Zheng-Qian Liu, Xin Zhou, Jun-Qi Liu, Lei Lu, Quan-Fa Han, Li-Ming Shang, Xin-Ping Ye, Tao Peng

**Affiliations:** 1 Department of Hepatobiliary Surgery, The First Affiliated Hospital of Guangxi Medical University, Nanning, 530021, Guangxi Zhuang Autonomous Region, People's Republic of China.; 2 Department of Colorectal and Anal Surgery, The First Affiliated Hospital of Guangxi Medical University, Nanning, 530021, Guangxi Zhuang Autonomous Region, People's Republic of China.; 3 Department of Hepatobiliary Surgery, The Third Affiliated Hospital of Guangxi Medical University, Nanning, 530031, Guangxi Zhuang Autonomous Region, People's Republic of China.; 4 Department of Pathology, First Affiliated Hospital of Guangxi Medical University, Nanning, Guangxi Zhuang Autonomous Region 530021, People's Republic of China.; 5 Department of Medical Oncology, First Affiliated Hospital of Guangxi Medical University, Nanning, Guangxi Zhuang Autonomous Region 530021, People's Republic of China.

**Keywords:** hepatitis B virus, hepatocellular carcinoma, overall survival, immunohistochemical, hepatectomy

## Abstract

**Objective:** The goal of our current study is to assess the immunohistochemical of p53, p21, nm23, and VEGF expression in hepatitis B virus (HBV)-related hepatocellular carcinoma (HCC) prognosis after hepatectomy, as well as the prospective molecular mechanisms of prognostic indicator.

**Methods:** There were 419 HBV-related HCC patients who were from southern China of Guangxi province and were used to evaluate the immunohistochemical expression for these biomarkers in prognosis. A genome-wide expression microarray dataset of HBV-related HCC were obtained from GSE14520.

**Results:** In our study, the expression of p53, p21, and nm23 in cancer tissues of patients with hepatitis B-related hepatocellular carcinoma did not affected the clinical outcome of 2 years, 5 years or overall. Patients with high expression of VEGF had a worse overall survival after 2 years of surgery than patients with low expression (adjusted *P*=0.040, adjusted HR = 1.652, 95% CI = 1.024-2.665). Survival analysis of VEGF in GSE14520 cohort also demonstrated that VEGF mRNA expression also significantly associated with HBV-related HCC OS (adjusted* P*=0.035, adjusted HR =1.651, 95% CI =1.035-2.634). The prospective molecular mechanisms by co-expression analysis suggested that VEGF might be correlated to regulation of cell proliferation, cell growth and apoptotic process, Rap1 signaling pathway, HIF-1 signaling pathway, PPAR signaling pathway, cell cycle. Whereas the GSEA suggested that VEGF might involve in the regulation of HIF and HIF1A pathway, and TP53 regulation pathway.

**Conclusion:** Our findings suggested that VEGF might be a prognostic indicator of HBV-related HCC, and we also identified the VEGF prospective molecular mechanisms through the whole genome co-expression and GSEA approaches.

## Introduction

Liver cancer is ranked as one of the top common malignant tumors around the globe. Merely in the year of 2012, over 780 thousand new cases of liver cancer were diagnosed each year in the world, with China 50% of the total number of cases 1. The annual incidence of liver cancer in China was 370,000 (27.29/100,000) with the death rate 310,000 (23.76/100,000), and ranked fourth in the third malignant tumor spectrum and the death spectrum respectively 2. Most (70% to 90%) liver cancers occurring worldwide are hepatocellular carcinoma (HCC) 3. There are many factors contributing to the development of HCC, including alcohol abuse, Aflatoxin-1 and hepatitis B virus (HBV), nonalcoholic fatty liver disease (NAFLD) and hepatitis C virus (HCV) infection 4, 5. In Guangxi, the male and female liver cancer mortality was 69.0/100,000 and 17.9/100,000 respectively, which was the highest fatality rate for male and female patients in China 6. Epidemiological studies showed that the major risk factors for liver cancer in Guangxi included three major risk factors: hepatitis viruses (especially HBV), aflatoxin (AFB) intake, and drinking water source pollution 7-10.

Even if the HCC patients after surgical resection or liver transplantation, the prognosis of HCC was still not satisfactory. The prognosis of liver cancer is affected by many clinical characteristics. Clinical characteristics such as vascular invasion, Barcelona Clinic Liver Cancer (BCLC) staging, tumor size, alpha-fetal protein (AFP), morphological and pathological features are traditionally the most important prognostic factors. Those related studies that had be conducted before had shown that the expression of p53 [also known as tumor protein p53 (TP53)], p21 [cyclin dependent kinase inhibitor 1A (CDKN1A)], nm23 [also known as nucleoside diphosphate kinase 1 (NME1)] and VEGF [also known as vascular endothelial growth factor A (VEGFA)] could reflect the prognosis of liver cancer by immunohistochemical techniques 11-14. p53 protein, a protein suppressing tumor, has response to diverse cellular stresses for regulating the expression of target genes, and thus induces senescence, apoptosis, cell cycle arrest, DNA repair, or changes in metabolism. p21, being a potent cyclin-dependent kinase inhibitor, performs as a regulator of cell cycle progression at G1 for it not only binding to but also inhibiting the activity of cyclin-cyclin-dependent kinase2 or cyclin-dependent kinase 4 complexes. VEGF, a heparin-binding protein, induces proliferation and migration of vascular endothelial cells and is vital to both physiological and pathological angiogenesis. This gene, whose expression is related to tumor stage and progression, is unregulated in many identified tumors. nm23, a suppressor for tumor metastasis, has regulation function towards a variety of cellular activities, which includes proliferation, differentiation, migration and apoptosis. Recent studies had shown the common understanding that the cell-cycle proteins could have interaction with nm23 and might function as modulators of the metastasis suppressor activity 15. Previous studies had shown different views about these immunohistochemical markers on prognosis, which might be the result of a different background in the research population of these studies. Guangxi is a highly exposed area of HBV. In this study, the expression of p53, p21, nm23, VEGF protein in tumor tissue of HBV-related HCC patients within Guangxi combined with other markers (such as AFP, BCLC stage, tumor size) were analyzed for estimating the prognostic value of patients after HCC resection.

## Methods

### Study population

The Ethics Committee of the First Affiliated Hospital of Guangxi Medical University had grant the approval for this study. We examined a total of 419 cases from the patients with HCC whose clinical characteristics from 2003 to 2013 were collected from the First Affiliated Hospital of Guangxi Medical University, Guangxi, China. All sample serological tests were positive for hepatitis B surface antigen (HBsAg) and histopathology were confirmed to be hepatocellular carcinoma. The tumor status was categorized by the BCLC staging system, and the liver function was identified according to the Child-Pugh classification. Portal vein tumor thrombus (PVTT) was identified in accordance with the previous study 16. The follow-up time of the patients was after surgery until death or the final follow-up which was conducted in September 2014.

### Immunohistochemical and scoring

All HCC samples were obtained during operation and stored right away at -80 C for further application. Tissue blocks prepared from HCC tissues were used to perform p53, p21, VEGF and nm23 immunohistochemistry (IHC). To be brief, all the specimens were cut off by formalin fixation and paraffin embedding, and triethylene-propyl triethoxysilane was processed into slices. The slices were routinely dewaxed and hydrated and washed in ethanol. Tissue immunohistochemical staining was conducted by the manufacturer's instructions. The sections were incubated by primary antibody (anti-p53, anti-p21, anti-nm23, anti-VEGF, at Vitrogen, Camarillo, CA) for 1 hour 37 °C. The working dilution of the primary antibody was 1:50. The slice was firstly PBS washed g for 15 minutes, then incubated with ENVISION+ rabbit/horseradish peroxidase for 45 minutes, and finally 15 minutes after Peroxidase. The positive and negative controls were performed on each section. Instead of the primary antibody, normal rabbit serum IgG was used as negative control. We conducted all experiments in duplicate.

p21 and p53 immunostaining was estimated quantitatively by counting the total number of positively stained nuclei per 10 high-power fields (×400 magnification) microscopically from the slides. We found that only nuclear staining was positive for p21 and p53. Established on the previously published criteria, positive staining of p21 and p53 was identified when >5% of tumor cells were stained 17, 18. The cases were considered positive to nm23 and VEGF protein expression if more than 10% of the tumors cells showed cytoplasm of tumor cells staining, as performed in previous studies 19, 20. Our research observed the stained sections under a light microscope (400×) (Olympus, Japan). Two independent pathologists confirmed the clinicopathological features of these patients. The mean percentage value of two cores was taken as the representative of one tumor, and discrepancies were resolved by consensus.

### Validation cohort at mRNA level and bioinformatics analysis

To verify the prognostic values of TP53, NME1, VEGFA and CDKN1A at mRNA level, GSE14520 (http://www.ncbi.nlm.nih.gov/geo/query/acc.cgi?acc=GSE14520), a genome-wide expression microarray dataset with HBV-related HCC was serve as validation cohort. The detailed procedure of data processing could be found in our previous studies 21. Then the prospective molecular mechanism of prognostic indicators of HBV-related HCC were investigated by gene set enrichment analysis (GSEA, http://software.broadinstitute.org/gsea/index.jsp) with the reference gene set from Molecular Signatures Database (MSigDB) gene sets: c2 gene set (c2.all.v6.1.symbols.gmt) and c5 gene set (c5.all.v6.1.symbols.gmt) 22, 23. In addition, genome-wide co-expression analysis to identified co-expression genes of the prognostic genes were used to investigated the potential biological processes and pathways that associated with prognostic genes in HBV-related HCC tumor tissues. The potential biological processes and pathways were identified by applying the Database for Annotation, Visualization and Integrated Discovery v6.8 (DAVID v6.8, https://david.ncifcrf.gov/home.jsp) 24, 25 and Biological Networks Gene Ontology tool (BiNGO) in Cytoscape version 3.6.1 26.

### Statistical analysis

Statistical analysis was to explore the relationship between the clinical parameters of gender, age, tumor size, number of tumors, pathologic of grade, serum level of AFP, and the 4 immunohistochemical markers by chi-square test. Survival analysis was assessed by the Kaplan-Meiercurve with the log-rank test. Overall survival (OS) was defined from the date of follow-up (September 1,2014). Univariate analysis, which was conducted to explore the relationship between clinical features and survival analysis, was applied to calculate the crude and those result with P<0.1 were fitted into the Cox proportional hazards regression model. Cox proportional hazards regression analysis was used to calculate adjusted hazard ratio (HR) and 95% confidence interval (CI) SPSS version 18.0 (SPSS, Inc., Chicago, IL, US) for Windows was applied for the statistical analyses. A value of P<0.05 was taken as statistically significant.

## Results

### Correlation analysis of immunohistochemical expression of p53, p21, nm23 and VEGF with clinicopathological characteristics

**Figure 1:**
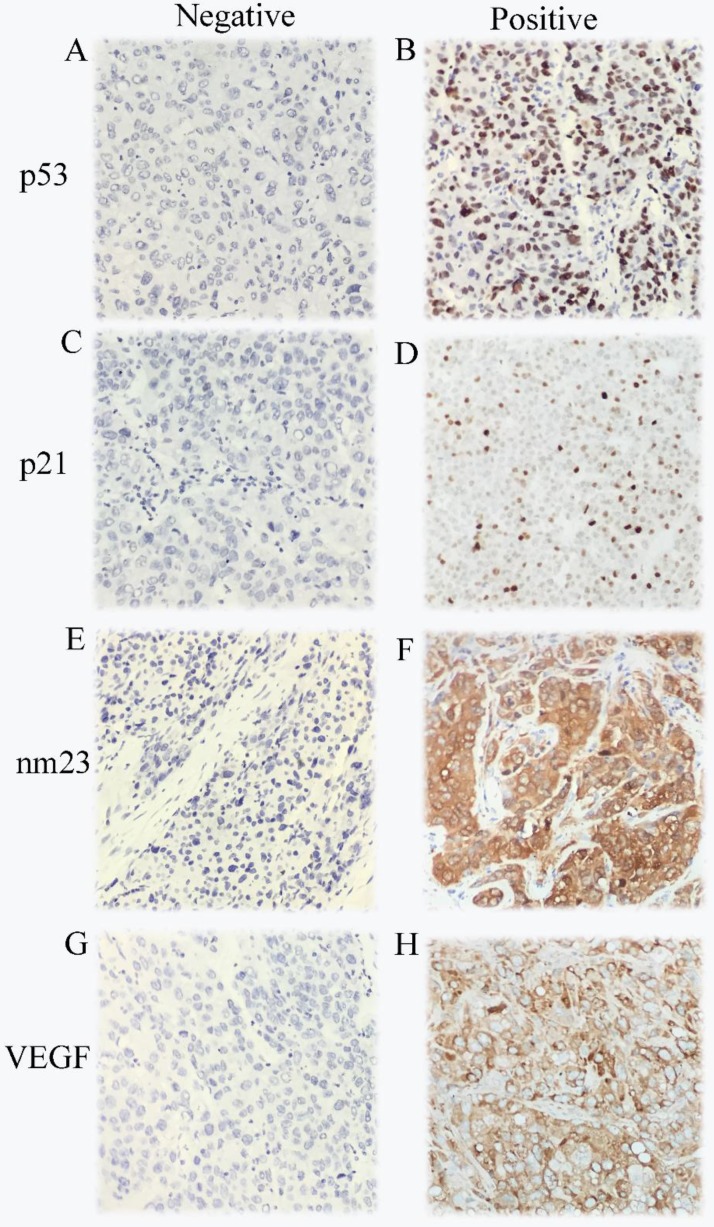
Immunohistochemical staining in HCC samples for p53 (A-B), p21 (C-D),nm23 (E-F) and VEGF (G-H).

The expression of p53, p21, nm23, and VEGF in the 419 HCC cases were analyzed by IHC. The immunostaining results showed that 255 cases were positive and 164 cases were negative for p53, 112 cases were positive and 307 cases were negative for p21, 376 cases were positive and 43 cases were negative for nm23, 320 cases were positive and 99 cases were negative for VEGF, respectively. Age, tumor size, cirrhosis, and antiviral therapy were significantly associated with p21 expression (χ^2^=5.722, *P*=0.017; χ^2^=4.358, *P*=0.037; χ^2^=9.576, *P*=0.002; χ^2^=12.564, *P*<0.001; respectively, **Table 1**). Antiviral therapy was closely correlative with nm23 expression (χ^2^=6.791, *P*=0.009). Race was considerably connected with p53 expression (χ^2^=5.014, *P*=0.025). Smoking status was significantly associated with VEGF expression (χ^2^=3.886, *P=*0.049). As demonstrated in **Figure 1**, p53 and p21 were mainly located in the nuclei of the cancer cell. nm23 while VEGF mainly in the cytoplasm of the cancer cell.

The median follow-up duration was 36.7 months, and the median survival time (MST) was 51 months. The distribution of clinical features in 419 patients with hepatocellular carcinoma was shown in **Table 2**. The gender, age, race, smoking status, BMI, AFP level, Child-Pugh, cirrhosis, and pathological grade were not notably associated with OS. However, the overall survival time was associated with alcohol status, BCLC status, portal vein tumor thrombus, antiviral therapy, tumor size, and tumor number (Log-rank *P* value for drinking status=0.043, for tumor size<0.001, for tumor number=0.001, for PVTT<0.001, for antivirus therapy=0.020). Drinking patients had a higher risk of death than those who do not drink (HR=1.335, 95% CI=1.066-1.770); patients with BCLC B or C stage had a higher risk of death than BCLC A stage patients (HR=1.880, 95% CI=1.281-2.758; HR=2.766, 95% CI=2.021-3.786; respectively); multiple tumor patients had a higher risk of death than single tumor patients (HR=1.642, 95% CI=1.219-2.212); patients with a tumor size greater than 5 cm had a higher risk of death than patients with tumor size≤5 cm (HR=1.981, 95%CI=1.416-2.770); patients with portal thrombosis had a higher risk of death than those without portal thrombosis (HR=2.801, 95% CI=2.025-3.875), and anti-HBV virus was less death risk than antiviral death (HR=0.675, 95%CI=0.483-0.945).

### Association between immunohistochemical markers and OS

**Figure 2:**
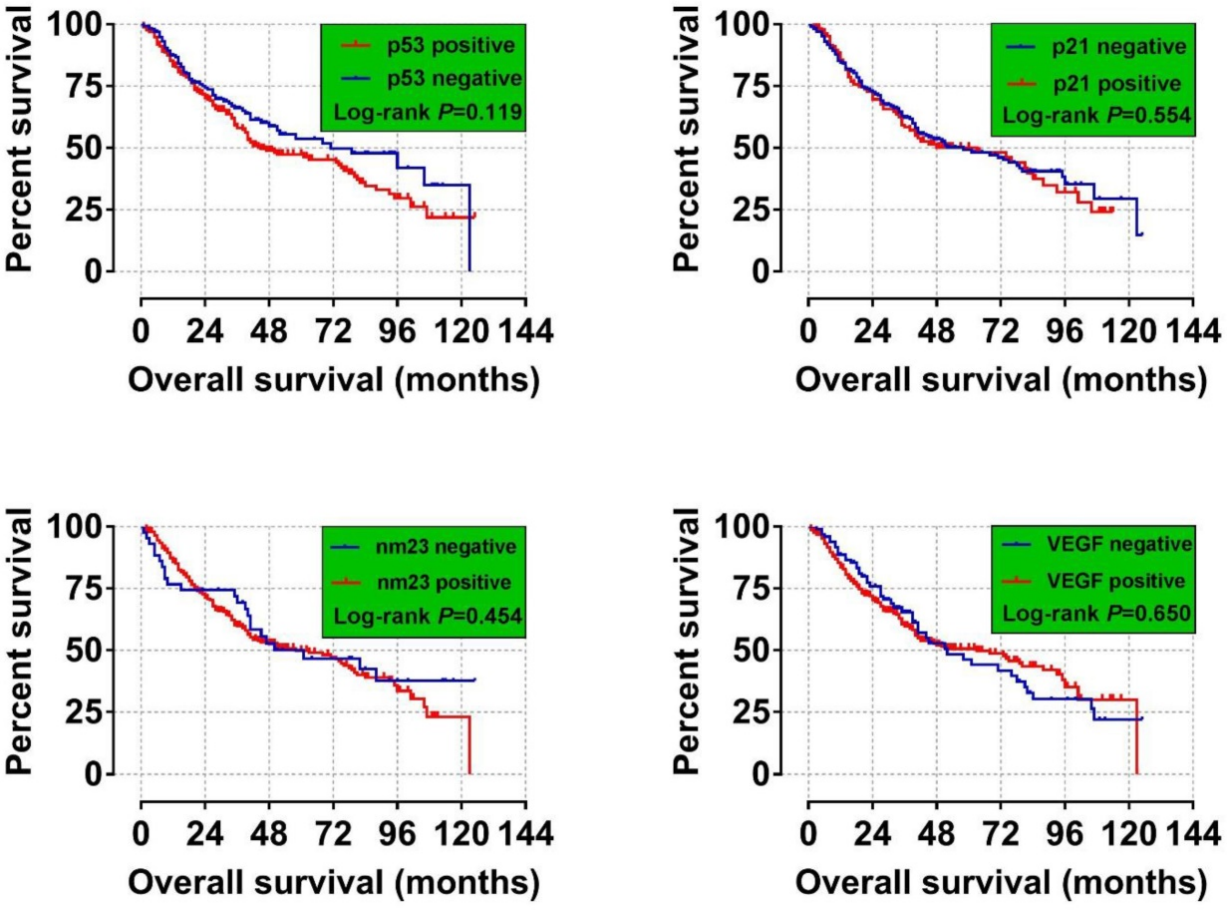
Overall Survival for HCC Patients with Different p53, p21, nm23, and VEGF Expression Statuses. (A) p53; (B) p21;( C) nm23; and (D) VEGF. It was no significant correlation between the four tumor markers and the long-term OS of HBV-associated hepatocellular carcinoma.

We analyzed the relationship between p53, p21, nm23 and VEGF expression and 2-year, 5-year and overall survival analysis. We found a significant difference in the 2-year survival time of patients with positive and negative VEGF (*P*= 0.040), VEGF-positive patients Death risk was higher than negative (HR= 1.652, 95% CI=1.024- 2.665) (**Table 3**). In current study we did not discover these four indicators significant related to the long-term OS of HBV-related HCC (**Figure 2 A-D**).

### Joint effects of immunohistochemical markers and AFP with OS

The combination of expression of p53, p21, nm23, and VEGF were divided into the relevant groups (**Table S1**) for assessing the prognostic value in HCC according to the associations between the immunohistochemical indicators and OS. As shown in **Table 4**, the 2-year survival analysis of joint effects were statistically different between group of score=2 (p53/VEGF) and group of score=0 (p53/VEGF) expression in 419 case tissues (*P*=0.047), p53/VEGF relative to p53/VEGF (+/+) was a protective factor for the prognosis of liver cancer (HR=0.450, 95% CI=0.205-0.988). The 2-year survival analysis of joint effects were statistically different between group of score=1 (P21/NM23/VEGF) and group of score=0 in 419 case tissues (*P*=0.043), group of score=1 relative to group of score=0 was a protective factor for the prognosis of liver cancer (HR=0.477, 95% CI=0.233-0.979). The 5-year survival analysis of joint effects were statistically different between group of score=1(p53/p21) and group of score=0 in 419 case tissues (*P*=0.027), group of score=1 relative to group of score=0 is a protective factor for the prognosis of liver cancer (HR=0.697, 95% CI=0.506-0.960). Among the 419 patients, group of the score=1 (P21/NM23/VEGF) and group of the score=3 was considerably different from the score =0 group (*P*=0.024, *P*=0.008, respectively), group of the score=1 and group of score =3 relative to group of score=0 were the protective factor for HCC (HR=0.503, 95%CI=0.0.277-0.913; HR=0.179,95%CI=0.050-0.638). Overall survival analysis showed that groups with a score of =3(P21NM23VEGF) had lower risk of death than those with a score of =0 (*P*=0.016, HR=0.311, 95%, CI=0.120-0.804).

Joint effects survival analysis indicated that the 2-year survival time of group of p53/AFP (+/high) were statistically different with group of p53/AFP (+ low) (*P* =0.038) in **Table 5**. The risk of death in group p53-VEGF (+/low) group was considerably lower than that in group p53/AFP (+/higher) (HR=0.584, 95% CI=0.352-0.969). The 2-year survival time of group of VEGF/AFP (-/low) were statistically different with group of VEGF/AFP (+/high) (*P* =0.027). The risk of death in group VEGF/AFP (-/low) group was notably lower than that in group VEGF/AFP (+/higher) (HR=0.444, 95% CI=0.217-0.910). The 5-year survival time of group of VEGF/AFP (+/low) were statistically different with group of VEGF/AFP (+/high) (*P*=0.026). The risk of death in group VEGF/AFP (+/low) group was remarkably lower than that in group VEGF/AFP (+/higher) (HR=0.518, 95% CI=0.291-0.923).

### Stratification analysis

**Figure 3:**
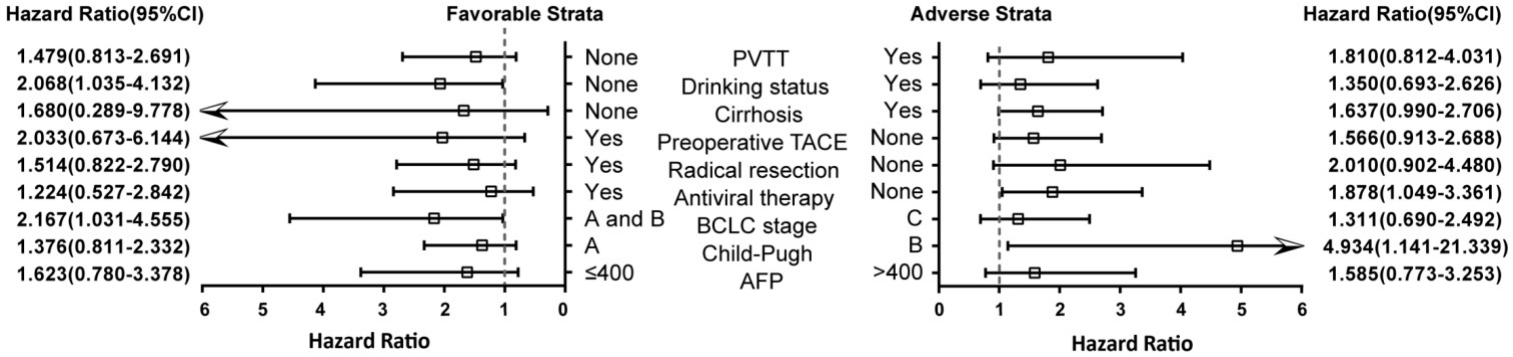
Stratification analysis of the association of VEGF with2-year OS of HBV-related HCC patients. Stratified by favorable and adverse strata.

We further studied VEGF expression with clinical features after 2 years of postoperative stratification analysis after adjusting for drinking status, BCLC stages, PVTT, radical hepatic resection and antiviral treatment (excluding the stratified factor in each stratum) (**Figure 3**). High VEGF expression could increase the risk of death in non-drinkers, BCLC stage A and B, non-Antiviral therapy and liver function Child B grade HCC patients (*P*=0.040, HR=2.068; *P*=0.041, HR=2.167; *P*=0.034, HR=1.878; *P*=0.033, HR=4.934; respectively).

### Validation cohort at mRNA level and bioinformatics analysis

**Figure 4:**
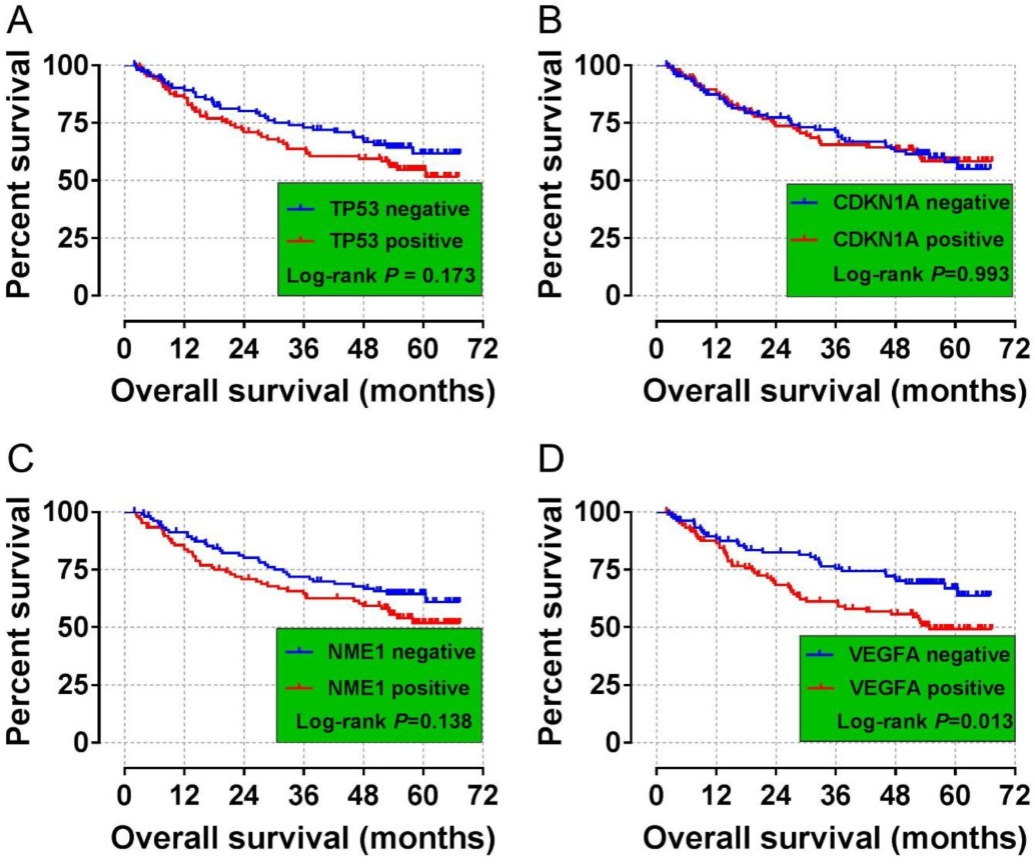
Survival curves for the GSE14520 analyses of HCC patients with different TP53, CDKN1A, NME1, and VEGFA mRNA expression levels. (A) Kaplan-Meier survival curves for OS for different TP53 expression levels. (B) Kaplan-Meier survival curves for the OS analyses of different CDKN1A expression levels. (C) Kaplan-Meier survival curves for the OS analyses of different NME1 expression levels. (D)Kaplan-Meier survival curves for the OS analyses of different VEGFA expression levels.

**Figure 5:**
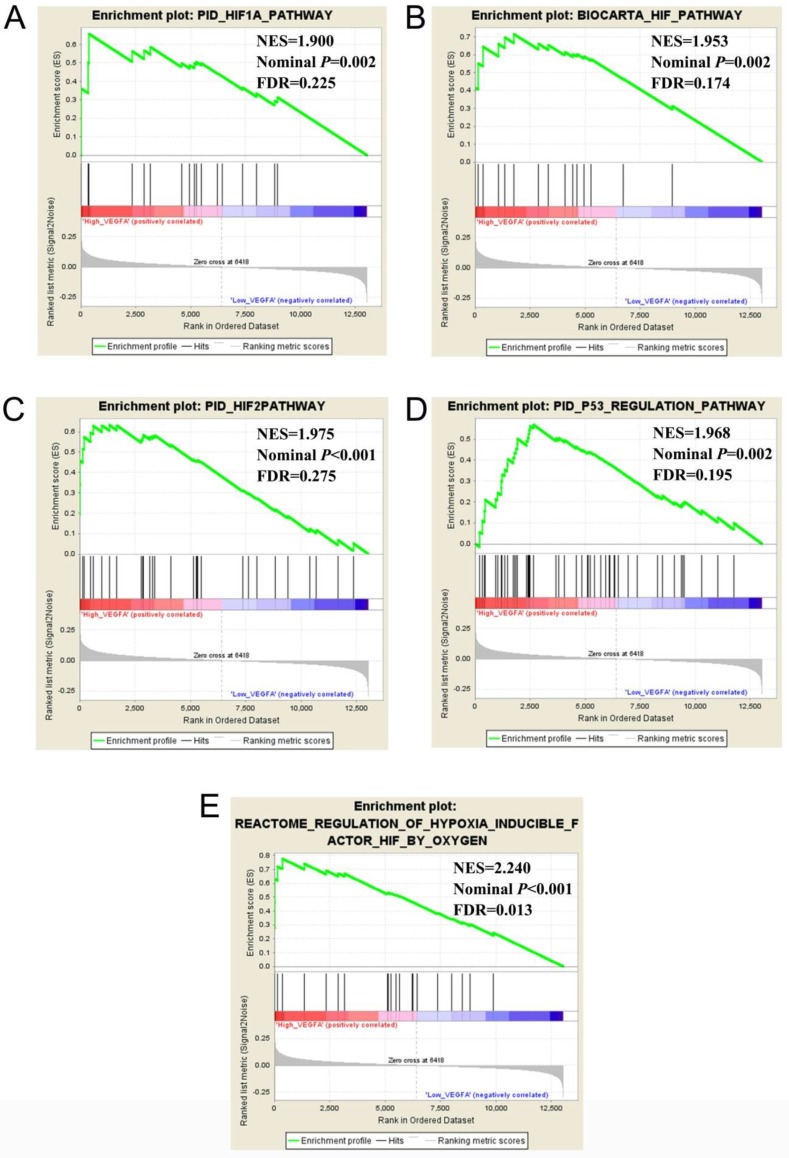
Comparative gene expression studies of HCC tumors and adjacent normal samples in GSE14520 dataset using GSEA. Notes: GSEA results of c2 (A-D) and c5 (E) Abbreviations: NES, Normalized enrichment score; FDR, false discovery rate.

The validation cohort of mRNA dataset were download from the GSE14520. A total of 212 HBV-related HCC were included into validation cohort, and the clinical parameters are summarized in **Table S2**. Survival analysis suggest that high VEGFA expression significantly linked to poor OS in patients with HBV-related HCC, whereas the other three genes were not showed the statistical significance (**Table 6, Figure 4 A-D**). Co-expression analysis of VEGFA in HBV-related HCC tumor tissues suggested that VEGFA and its co-expression genes were significant correlated to regulation of cell proliferation, cell growth and apoptotic process, G1/S transition of mitotic cell cycle, cellular response to hypoxia, protein binding, enzyme binding, protein complex assembly, DNA damage checkpoint, Rap1 signaling pathway, HIF-1 signaling pathway, PPAR signaling pathway, cell cycle, biosynthesis of amino acids, and cellular response to hypoxia (Table S3, Figure S1), which were based on the analysis of Gene ontology (GO) and Kyoto Encyclopedia of Genes and Genomes (KEGG) in DAVID v6.8. Prospective molecular mechanisms revealed that high VEGFA expression might participate in the following biological processes and pathways: regulation of transcription from RNA polymerase II promoter in response to hypoxia, regulation of Hypoxia-inducible Factor (HIF) by Oxygen, HIF and HIF1A pathway, and TP53 regulation pathway (**Figure 5A-E**).

## Discussion

HCC is a highly malignant tumor with poor prognosis. Although the treatment of HCC has important clinical outcomes over the past few decades, the prognosis of HCC patients is still unsatisfactory and has a higher rate of local recurrence and/or distant metastasis. Unfortunately, the prognostic indicators that can guide the treatment of hepatocellular carcinoma are limited, so the survival rate of patients with malignant tumors after surgical resection requires clinicians to participate in the active treatment of relapse and to study the biological and clinicopathological features that reflect tumor behavior.

As we all known, p53, p21, nm23 and VEGF are important biomarkers for diagnosis and assessment the prognosis of HCC. IHC analysis revealed that p53 gene mutations were correlated with the p53 expression and most of HCC tumor tissue with p53 mutations exhibited positive staining for p53 protein 27. HCC patients with p53 mutation and up regulated expression in tumor tissue had a shorter OS than those with wild type p53 and low/undetectable p53 expression 28. However, Chai Y et al. reported p53 expression was not related to cancer characteristics 9. Prior studies indicated p21 expression was a predictor for clinical performance of patients with HCC, those who had a high p21expression predicted a better survival 17, 29. However, the predictor value of p21 in HCC patients was affected by HBV proteins and p53 expression 30-33. Although p53 expression did not link to OS of HBV-related HCC patients in this study, the patients with positive for p53 expression had higher HR than those negative for p53 expression. p21 expression was associated with some clinical features but not with prognosis of HCC patients. Combined analysis showed p53 and p21 expression levels were associated with 5-year OS. Recent study demonstrated that HCC patients with high VEGF isoforms expression was associated with shorter RFS and poor prognosis 34. In this study, no significantly difference were found among p53, p21, nm23, VEGF expression level and clinical outcomes of HBV-related HCC patients. We applied different combined analysis in groups using different combinations and our results suggested combination of p53, p23 and VEGF expression might be a good predictor for latter recurrence of HCC patients after hepatectomy. Although previous studies did not apply the combination of these genes as a method of evaluation, our research provided a good research strategy. Further, we needed to collect multiple centers and a larger number of samples to validate our results.

AFP is one of the most commonly used biomarkers in the diagnosis and evaluation of clinical outcomes of HBV related HCC 5, 35. However, there is still some controversy over the prognostic value of AFP 12, 14, 36, 37. In this study, we attempted to perform a joint analysis of AFP and immunohistochemical markers to explore whether such conjoint analysis could improve the predictive efficacy of clinical outcomes. In this report, 4 immunohistochemical indicators were combined with AFP level for analysis of clinical outcomes of HBV-related HCC patients. We found that p53 positive patients with low levels of AFP had better 2 years survival than those who both high levels of AFP and p53 positive. Compared to patients with VEGF positive and high level of AFP, VEGF negative and low level of AFP patients had a good two years of survival, and patients with VEGF positive and low level of AFP had a good five-year survival time. AFP was not a strong prognostic marker despite the fact that serum AFP level above 400 ng/ml would indicate poor overall survival time after hepatectomy in patients with HBV-associated HCC 38. Some studies reported that combined analysis enhanced diagnosis and prognosis value of AFP in HCC 39, 40. Our results suggested joint analysis of AFP, p53 and VEGF might be performed for the prediction of the clinical outcomes of HBV-related HCC patients in Guangxi.

As shown in previous studies 4, 7, 41, 42, although four indicators had an effect on the prognosis of patients with hepatocellular carcinoma after surgery, joint analysis was less. This research attempted to study the relationship between the combination of four immunohistochemical indicators and the prognosis. We found that the patients with three proteins combined with P21, NM23, and VEGF, the group of score =1 and group of score =3 had a longer survival time in 5 years than group of score =0. Any protein that gives a score greater than 0 was a protective factor for the prognosis. However, for 2 years and overall survival, the statistical P values were near 0.05, and perhaps increasing the sample size might show statistical difference.

Previous study had shown that VEGFA expression could be activated by transcription of various transcription factors, including Sp1, NFκB, AP1 and HIF-1α. HIF-1α could inhibit VEGFA expression, whereas VEGF-mediated upregulation of IL-6 triggers the progression of hemangioma cells 43. We found through the functional enrichment of VEGFA and its co-expression related genes that VEGFA affects tumors basic cell states by participating in the regulation of cell proliferation, cell cycle, apoptosis.

Our research had certain limitations that needed to be recognized. First, because of the small sample size, the prognosis of many immunohistochemical markers did not reach statistical significance. Second, our sample size was not large enough to verify the impact of rare levels on OS in stratified analysis. Third, our samples came from HBV positive HCC in Guangxi, and our results required larger samples and multicenter validation. Fourth, since the molecular mechanism of VEGFA in this study was explored by GSEA, the validation of *in vitro* and *in vivo* experiments was lacking. Therefore, our results still need to be experimentally verified in future study.

Despite these limitations, our study was the first to predict the prognosis of HBV-related HCC using four immunohistochemical indicators and AFP assessment. Our results suggested that the four immunohistochemical indicators had some clinical value in predicting the prognosis of HCC. The prognostic value of the four immunohistochemical indicators and AFP in HBV-related HCC patients could be enhanced using combined and stratified analysis.

## Conclusion

In conclusion, our findings demonstrated that expression of VEGF may play the role as a prognostic indicator for patients with HBV-related HCC. The prospective molecular mechanism of VEGF might involve in the biological processes and pathways of hypoxia, cell cycle, cell apoptosis, cell proliferation and DNA damage checkpoint, which were importance for the base status of normal cells.

## Supplementary Material

Supplementary figures and tables.

## Figures and Tables

**Table 1:** Correlation analysis of immunohistochemical expression of p53, p21, nm23 and VEGF with clinicopathological data.

		p53				p21				nm23				VEGF				
Variables	Number	Negative	Positive	χ^2^	*p*	Negative	Positive	χ^2^	*p*	Negative	Positive	χ^2^	*p*	Negative	Positive	χ^2^	*p*	
**Gender**																		
Male	377	149	228	0.230	0.631	274	103	0.670	0.413	40	337	0.493	0.482	85	292	2.437	0.119	
Female	42	15	27			33	9			3	39			14	28			
**Age**																		
≤46	229	83	146	1.778	0.182	157	72	5.722	**0.017**	20	209	1.282	0.258	57	172	0.447	0.504	
>46	190	81	109			150	40			23	167			42	148			
**Race**																		
Han	261	113	148	5.014	**0.025**	189	72	0.259	0.611	32	229	3.000	0.083	62	199	0.006	0.937	
Minority	158	51	107			118	40			11	147			37	121			
**Smoking status**																		
No	270	109	161	0.482	0.488	195	75	0.425	0.514	29	241	0.189	0.664	72	198	3.886	**0.049**	
Yes	149	55	94			112	37			14	135			27	122			
**Drinking status**																		
No	250	101	149	0.413	0.521	180	70	0.510	0.475	27	223	0.194	0.659	60	190	0.048	0.827	
Yes	169	63	106			127	42			16	153			39	130			
**BMI**																		
≤25	348	135	213	0.104	0.747	249	99	3.095	0.079	38	310	0.963	0.327	78	270	1.677	0.195	
>25	71	29	42			58	13			5	66			21	50			
**AFP(ng/ml)**^ a^																		
≤400	214	92	122	2.302	0.129	164	50	2.007	0.157	23	191	0.275	0.600	55	159	0.841	0.359	
>400	175	62	113			123	52			16	159			38	137			
**Child pugh**^ b^																		
A	337	127	209	1.503	0.220	248	88	0.425	0.514	31	305	1.371	0.242	77	259	0.848	0.357	
B	56	26	30			39	17			8	48			16	40			
**BCLC stage**																		
A	239	93	145	0.136	0.934	174	64	0.693	0.707	30	208	3.464	0.177	55	183	1.170	0.557	
B	69	28	41			53	16			4	65			14	55			
C	111	42	69			79	32			9	102			30	81			
**No. of tumors**																		
Single (n=1)	308	119	189	0.124	0.725	227	81	0.111	0.739	36	272	2.566	0.109	76	232	0.707	0.400	
Multiple (n>1)	111	45	66			80	31			7	104			23	88			
**Tumor size**																		
≤5cm	138	58	80	0.721	0.396	110	28	4.358	**0.037**	17	121	0.945	0.331	31	107	0.154	0.694	
>5cm	281	106	175			197	84			26	255			68	213			
**Cirrhosis**																		
No	50	15	34	1.640	0.200	27	22	9.576	**0.002**	7	42	0.962	0.327	9	40	0.868	0.351	
Yes	369	148	221			280	89			36	333			90	279			
**Pathological grade**^ c^																		
Well	24	14	10	3.787	0.052	22	2	4.306	**0.038**	3	21	0.196	0.658	7	17	0.437	0.509	
Moderately/Poorly	340	130	210			246	94			33	307			79	261			
**PVTT**																		
No	352	141	211	1.500	0.827	258	94	0.070	0.999	37	315	2.087	0.720	79	273	5.325	0.256	
Yes	67	3	8			8	3			1	10			1	10			
**Radical resection^d^**																		
No	169	89	149	0.491	0.484	172	66	0.420	0.517	24	214	0.034	0.853	63	175	2.644	0.104	
Yes	238	69	100			127	42			18	151			33	136			
**Antiviral therapy**																		
No	276	103	173	1.127	0.288	187	89	12.564	**<0.001**	36	240	6.791	**0.009**	65	211	0.003	0.959	
Yes	143	61	82			120	23			7	136			34	109			

^a^ information regarding AFP level was unavailable for 30 patients; ^b^ information regarding child-pugh was unavailable for 26 patients; ^c^ information regarding pathological grade was unavailable for 55 patients; ^d^: information regarding radical resection was unavailable for 12 patients. HBV, hepatitis B virus; HCC, hepatocellular carcinoma; BMI, body mass index; AFP, α-fetoprotein; BCLC, Barcelona Clinic Liver Cancer; PVTT, portal vein tumor thrombus.

**Table 2:** Clinical features of the patients with HBV-related HCC.

Variable	Patients (n=419)(%)	MST (months)	Log-rank *P*	HR(95% CI)
**Gender**				
Male	377	51	0.277	1
Female	42	80		0.749(0.442-1.268)
**Age(year)**				
≤46	229	61	0.974	1
>46	190	51		1.005(0.758-1.331)
**Race**				
Han	261	51	0.875	1
Minority	158	52		1.023(0.764-1.371)
**Smoking status**				
No	270	71	0.106	1
Yes	149	41		1.267(0.949-1.692)
**Drinking status**				
No	250	76	0.043	1
Yes	169	41		1.335(1.006-1.770)
**BMI**				
≤25	348	52	0.918	1
>25	71	51		0.981(0.683-1.410)
**AFP(ng/ml)^a^**				
≤400	214	63	0.114	1
>400	175	42		1.265(0.943-1.697)
**Child-pugh^b^**				
A	337	58	0.181	1
B	56	34		1.291(0.877-1.900)
**BCLC stage**				
A	239	123	<0.001	1
B	69	95		1.880(1.281-2.758)
C	111	29		2.766(2.021-3.786)
**No. of tumors**				
Single(n=1)	308	63	0.001	1
Multiple(n>1)	111	34		1.642(1.219-2.212)
**Tumor size**				
≤5cm	138	123	<0.001	1
>5cm	281	40		1.981(1.416-2.770)
**Cirrhosis**				
No	50	88	0.191	1
Yes	369	51		1.358(0.854-2.158)
**Pathological grade^c^**				
Well	24	79	0.473	1
Moderately/Poorly	340	51		1.276(0.651-2.499)
**PVTT**				
No	352	47	<0.001	1
Yes	67	40		2.801(2.025-3.875)
**Radical resection^d^**				
No	169	41	0.115	1
Yes	238	73		1.254(0.944-1.667)
**Antiviral therapy**				
No	276	42	0.020	1
Yes	143	NA		0.675(0.483-0.945)

Notes: ^a^ information regarding AFP level was unavailable for 30 patients; ^b^ information regarding child-pugh was unavailable for 26 patients; ^c^ information regarding pathological grade was unavailable for 55 patients; ^d^ information regarding radical resection was unavailable for 12 patients. HBV, hepatitis B virus; HCC, hepatocellular carcinoma; BMI, body mass index; AFP, α-fetoprotein; BCLC, Barcelona Clinic Liver Cancer; PVTT, portal vein tumor thrombus; MST, median survival time; HR, hazard ratio; CI, confidence interval; NA, not available.

**Table 3:** Survival analysis between immunohistochemical expression of p53, p21, nm23 and VEGF with 2-year, 5-year and overall survival.

Variable	Number (n=419)	2-year OS *p*-value^ a^	2-yearOS HR(95% CI)^ a^	5-year *p*-value^ a^	5-yearOS HR(95% CI)^ a^	Overall survival *p*-value^ a^	Overall survival HR(95% CI)^ a^
**P53**							
-	164		1.000				1.000
+	255	0.731	1.070(0.729-1.569)	0.410	1.142(0.832-1.568)	0.284	1.178(0.873-1.591)
**p21**							
-	307		1.000				1.000
+	112	0.722	1.077(0.715-1.623)	0.803	0.958(0.686-1.340)	0.997	0.999(0.732-1.365)
**nm23**							
-	43		1.000				1.000
+	376	0.779	0.913(0.484-1.722)	0.650	0.895(0.554-1.445)	0.971	0.992(0.631-1.558)
**VEGF**							
-	99		1.000				1.000
+	320	**0.040**	1.652(1.024-2.665)	0.152	1.310(0.905-1.896)	0.130	1.303(0.925-1.834)

Notes:^ a^Adjusted for drinking status, BCLC stages, PVTT, radical hepatic resection and adjuvant antiviral treatment. OS, overall survival; HR, hazard ratio; CI, confidence interval.

**Table 4:** Joint effects analysis of expression of p53, p21, nm23 and VEGF with 2-year, 5-year and overall survival.

Variable	Number (n=419)	2-year OS P-value	2-year OS HR(95% CI)	5-year OS P-value	5-year OS HR(95% CI)	Overall survival *P*-value	Overall survival HR(95% CI)
**p53/p21**							
0	176	0.061	1.000	**0.036**	1.000	0.187	1.000
1	210	0.138	0.743(0.502-1.100)	**0.027**	0.697(0.506-0.960)	0.089	0.771(0.571-1.041)
2	33	0.200	1.500(0.807-2.788)	0.557	1.171(0.692-1.981)	0.898	1.034(0.619-1.726)
**p53/nm23**							
0	32	0.607	1.000	0.493	1.000	0.364	1.000
1	234	0.529	1.289(0.585-2.837)	0.750	1.095(0.627-1.910)	0.543	1.174(0.700-1.970)
2	153	0.858	1.077(0.476-2.438)	0.708	0.895(0.500-1.600)	0.819	0.939(0.547-1.612)
**p53/VEGF**							
0	202	0.125	1.000	0.334	1.000	0.229	1.000
1	171	0.994	0.998(0.680-1.465)	0.291	0.840(0.608-1.161)	0.261	0.839(0.618-1.139)
2	46	**0.047**	0.450(0.205-0.988)	0.204	0.713(0.423-1.201)	0.122	0.675(0.410-1.111)
**p21/nm23**							
0	31	0.317	1.000	0.237	1.000	0.468	1.000
1	288	0.192	0.639(0.326-1.252)	0.094	0.641(0.380-1.079)	0.266	0.748(0.449-1.247)
2	100	0.536	0.798(0.390-1.631)	0.247	0.717(0.409-1.259)	0.553	0.847(0.490-1.464)
**p21/VEGF**							
0	225	0.421	1.000	0.189	1.000	0.319	1.000
1	177	0.263	0.802(0.545-1.18)	0.818	0.964(0.705-1.318)	0.800	0.962(0.713-1.297)
2	17	0.385	0.635(0.228-1.771)	0.068	0.389(0.141-1.073)	0.131	0.578(0.284-1.178)
**nm23/VEGF**							
0	32	0.141	1.000	0.321	1.000	0.289	1.000
1	299	0.701	0.872(0.435-1.751)	0.687	0.894(0.518-1.542)	0.868	1.046(0.617-1.773)
2	88	0.133	0.538(0.240-1.208)	0.222	0.675(0.359-1.269)	0.411	0.778(0.427-1.416)
**p53/p21/nm23**							
0	23	0.201	1.000	0.104	1.000	0.388	1.000
1	170	0.690	0.849(0.379-1.899)	0.462	0.797(0.436-1.458)	0.817	0.932(0.516-1.685)
2	196	0.335	0.674(0.302-1.504)	0.097	0.600(0.329-1.096)	0.329	0.747(0.416-1.342)
3	30	0.570	1.313(0.513-3.362)	0.957	1.021(0.49-2.126)	0.912	1.041(0.507-2.138)
**p53/p21/VEGF**							
0	135	0.543	1.000	0.303	1.000	0.302	1.000
1	198	0.413	0.840(0.553-1.276)	0.081	0.735(0.519-1.039)	0.122	0.770(0.553-1.073)
2	81	0.158	0.667(0.381-1.17)	0.218	0.763(0.497-1.173)	0.257	0.791(0.527-1.187)
3	5	0.933	1.064(0.250-4.529)	0.376	0.523(0.125-2.195)	0.184	0.440(0.131-1.479)
**p21/nm23/VEGF**							
0	22	0.156	1.000	**0.028**	1.000	0.067	1.000
1	222	**0.043**	0.477(0.233-0.979)	**0.024**	0.503(0.277-0.913)	0.071	0.581(0.322-1.048)
2	160	0.058	0.498(0.242-1.024)	0.085	0.591(0.325-1.075)	0.223	0.691(0.381-1.253)
3	15	0.061	0.282(0.075-1.061)	**0.008**	0.179(0.050-0.638)	**0.016**	0.311(0.120-0.804)
**p53/nm23/VEGF**							
0	25	0.198	1.000	0.448	1.000	0.300	1.000
1	191	0.792	1.121(0.479-2.624)	0.872	1.052(0.569-1.944)	0.556	1.194(0.662-2.153)
2	161	0.846	1.089(0.460-2.578)	0.668	0.871(0.463-1.638)	0.941	0.977(0.535-1.786)
3	42	0.167	0.447(0.143-1.400)	0.375	0.705(0.325-1.527)	0.459	0.757(0.362-1.582)
**p53/p21/nm23/VEGF**							
0	17	0.734	1.000	0.283	1.000	0.390	1.000
1	137	0.378	0.675(0.282-1.618)	0.347	0.721(0.364-1.426)	0.593	0.831(0.421-1.638)
2	185	0.261	0.613(0.261-1.439)	0.092	0.562(0.287-1.099)	0.261	0.683(0.352-1.328)
3	76	0.205	0.549(0.217-1.388)	0.240	0.650(0.316-1.335)	0.450	0.759(0.372-1.551)
4	4	0.407	0.405(0.048-3.428)	0.136	0.207(0.026-1.645)	0.092	0.262(0.055-1.245)

Notes:^ a^ Adjusted for drinking status, BCLC stages, PVTT, radical hepatic resection and adjuvant antiviral treatment. OS, overall survival; HR, hazard ratio; CI, confidence interval; NA, not available.

**Table 5:** Joint effects analysis between 4 protein and AFP with 2-year, 5-year and overall survival.

Variables	Number (n=389)^a^	2-year OS *p*-value^b^	2-year OS HR(95% CI)^ b^	5-year OS *p*-value^b^	5-year OS HR(95% CI)^ b^	Overall survival *p*-value^b^	Overall survival HR(95% CI)^ b^
**p53/AFP**							
+/high	113	0.222		0.298		0.343	
-/low	92	0.438	0.812(0.480-1.374)	0.203	0.750(0.481-1.169)	0.248	0.777(0.506-1.192)
+/low	122	**0.038**	0.584(0.352-0.969)	0.110	0.724(0.487-1.075)	0.249	0.804(0.554-1.165)
-/high	62	0.345	0.760(0.429-1.345)	0.147	0.693(0.423-1.137)	0.097	0.668(0.415-1.075)
**p21/AFP**							
+/high	52	0.504		0.701		0.859	
-/low	164	0.294	0.734(0.411-1.309)	0.524	0.857(0.534-1.376)	0.833	0.952(0.604-1.501)
+/low	50	0.530	0.790(0.378-1.650)	0.463	0.803(0.446-1.444)	0.902	1.034(0.603-1.774)
-/high	123	0.958	1.015(0.575-1.794)	0.911	1.028(0.638-1.656)	0.635	1.118(0.706-1.770)
**nm23/AFP**							
+/high	159	0.424		0.647		0.883	
-/low	23	0.373	0.581(0.176-1.917)	0.917	0.962(0.466-1.986)	0.908	1.040(0.535-2.023)
+/low	191	0.205	0.769(0.513-1.154)	0.271	0.827(0.590-1.160)	0.536	0.903(0.655-1.246)
-/high	16	0.600	1.258(0.533-2.971)	0.708	1.145(0.563-2.330)	0.770	1.106(0.563-2.171)
**VEGF/AFP**							
+/high	137	0.100		0.197		0.130	
-/low	55	**0.027**	0.444(0.217-0.910)	0.133	0.672(0.400-1.129)	0.291	0.778(0.489-1.239)
+/low	38	0.164	0.605(0.298-1.228)	0.094	0.598(0.328-1.092)	**0.026**	0.518(0.291-0.923)
-/high	159	0.201	0.756(0.493-1.161)	0.155	0.772(0.540-1.103)	0.182	0.792(0.562-1.115)

Notes: ^a^ information regarding AFP level was unavailable for 30 patients (n=389); HR, hazard ratio; CI, confidence interval. ^b^ Adjusted for drinking status, BCLC stages, PVTT ,radical hepatic resection and adjuvant antiviral treatment. OS, overall survival; HR, hazard ratio; CI, confidence interval; NA, not available.

**Table 6:** Long-term survival analysis of mRNA expression of TP53, CDKN1A, NME1 and VEGFA in 212 cases of HBV-related HCC in GEO database 14520 data set

Gene expression	Patients(n=212)	MST (months)	Crude HR (95% CI)	Crude P	Adjusted HR (95% CI)	Adjusted P^a^
**TP53**						
Low	106	NA	1		1	
High	106	NA	1.352(0.874-2.093)	0.175	1.081(0.686-1.703)	0.737
**CDKN1A**						
Low	106	NA	1		1	
High	106	NA	1.002(0.650-1.545)	0.993	1.058(0.674-1.662)	0.806
**NME1**						
Low	106	NA	1		1	
High	106	NA	1.389(0.898-2.149)	0.140	1.316(0.842-2.057)	0.229
**VEGFA**						
Low	106	NA	1		1	
High	106	54	1.735(1.115-2.699)	0.015	1.651(1.035-2.634)	0.035

Notes: ^a^ Adjusted for AFP, BCLC stages, number of tumors, tumor size and cirrhosis. p53 also known as tumor protein p53 (TP53), p21 also known as cyclin dependent kinase inhibitor 1A (CDKN1A), nm23also known as nucleoside diphosphate kinase 1 (NME1) and VEGF also known as vascular endothelial growth factor A (VEGFA). HBV, hepatitis B virus; HCC, hepatocellular carcinoma; BCLC, Barcelona Clinic Liver Cancer; AFP, α-fetoprotein; MST, median survival time; OS, overall survival; HR, hazard ratio; CI, confidence interval; NA, not available.
