# Supplementary material for: Comprehensive investigation of p53, p21, nm23, and VEGF expression in hepatitis B virus-related hepatocellular carcinoma overall survival after hepatectomy
Source: J Cancer. 2020 Jan 1;11(4):906–18. doi: 10.7150/jca.33766 (PMC6959013; doi:10.7150/jca.33766)
Supplement: Supplementary file 1 — Supplementary figures and tables. [file jcav11p0906s1.pdf]

Supplementary material

Table S1. Grouping information for the combination among the expression of p53,p21,nm23,and VEGF.

Group	Score=0	Score=1	Score=2	Score=3	Score=4
p53/p21	+/-	+/+;-/-	-/+		
p53/nm23	+/-	+/+;-/-	-/+		
p53/VEGF	+/+	+/-;-/+	-/-		
p21/nm23	-/-	-/+;+/-	+/+		
p21/VEGF	-/+	+/+;-/-	+/-		
nm23/VEGF	-/+	+/+;-/-	+/-		
p53/p21/nm23	+/-/-	-/-/-;+/+/-;+/-/+	+/+/+;-/-/+;-/+/-	-/+/+	
p53/p21/VEGF	+/-/+	-/-/+;+/+/+;+/-/-	+/+/-;-/-/-;-/+/+	-/+/-	
p53/nm23/VEGF	+/-/+	-/-/+;+/+/+;+/-/-	+/+/-;-/-/-;-/+/+	-/+/-	
p21/nm23/VEGF	-/-/+	+/-/+;-/+/+;-/-/-	-/+/-;+/-/-;+/+/+	+/+/-	
p53/p21/nm23/VEGF	+/-/-/+	-/-/-/+;+/+/-/+;<br>+/-/+/+;+/-/-/-	-/+/-/+;+/+/+/+;+/-/+/-;<br>-/-/-/-;-/-/+/+;+/+/-/-	+/+/+/-;-/-/+/-;<br>-/+/-/-;-/+/+/+	-/+/+/-

Notes: According to previous studies show the role of these proteins,defined p53 positive =0, p53 negative =1, p21 positive =1, p21 negative =0, nm23 positive =1, nm23 negative =0, VEGF positive =0, VEGF negative =1.

Table S2. Clinical parameters of 212 HBV-related HCC in GSE14520 database

Variables		Patients(n=212)	OS			
			No. of events	MST(months)	HR (95% CI)	*P*
Age(year)						
	≤60	175	69	NA	1	
	>60	37	13	NA	0.864(0.478-1.564)	0.63
Gender						
	Female	29	8	NA	1	
	Male	183	74	NA	1.704(0.821-3.534)	0.152
Multinodular						
	Single	167	59	NA	1	
	Multiple	45	23	47	1.607(0.992-2.604)	0.054
Tumor Size[₤]						
	≤5	137	46	NA	1	
	>5	74	36	53	1.975(1.274-3.060)	**0.002**
Cirrhosis						
	NO	17	2	NA	1	
	Yes	195	80	NA	4.335(1.065-17.638)	**0.041**
BCLC stage[£]						
	0	20	2	NA	1	
	A	74	48	NA	4.119(1.001-16.951)	**0.05**
	B	22	12	46	8.992(2.005-40.320)	0.004
	C	27	20	13	18.993(4.419-81.632)	<0.001
AFP(ng/ml)[Ψ]						
	≤300	115	39	NA	1	
	>300	94	43	NA	1.546(1.002-2.385)	**0.049**

Notes: [₤] information regarding Tumor Size was unavailable for 1 patients; [£] information regarding BCLC stage was unavailable for 69 patients; [Ψ] information regarding AFP level was unavailable for 3 patients;

Table S3. GO and KEGG enrichment results of VEGF and its co-expression genes

Category	Term	Count	%	P Value	Genes
**GOTERM_MF_DIRECT**	GO:0005515~protein binding	562	0.392694	5.46E-13	LDHA, XRCC3, XRCC2, EHHADH, RUSC1, PPP2R5D, RUSC2, VPS52, CXORF40A, INTS3, MYLIP, KIAA0319L, SSR1, TMEM140, CUL3, CDCA8, HTR1B, CLK2, INTS7, INCENP, MAP3K9, SERPINE1, LSM2, LSM1, DDAH2, PCBD1, MPDZ, CRYAB, PTBP1, ESPL1, PTPRO, MARK2, GRB10, ZNF783, SPAG4, UBL4A, MAPK7, MYBBP1A, ADAMTS5, SH3GL1, HSD17B10, KIAA1109, PPP2R3B, NFKBIE, ERBB3, HFE, HSPA1A, NAGK, VPS72, PPP1R3C, IL23A, RAC1, MKI67, HGD, UBE2L6, TMEM110, GAS1, DVL1, SYNE1, UPF3B, WDR61, FNBP4, H3F3A, HGS, TNK1, POP7, RERE, ELF3, MARCKSL1, GRIP1, EZH2, BNIP3, RNF187, PDIA4, RASSF7, DAXX, DGCR14, GTSE1, RTN3, IL4R, SPG21, USP11, MAGEH1, ANGPT2, SLC22A1, FMNL1, CDC6, ARHGEF2, SH3PXD2A, CLIC1, MMP15, WAS, TTF2, CARD10, KLHDC3, TAF11, INHBB, INHBA, DOK2, MAST2, USP22, THOC2, TBC1D9, MKNK2, HK2, FPR1, FHL2, ITM2C, SF3B4, SF3B2, NPDC1, SORBS3, EXOC4, WIPF2, SERPINC1, DHX16, PPP3CA, ARMCX1, EXOC2, RASA1, FLRT3, SSRP1, SHMT2, BRD2, BRD3, SELL, PAK1IP1, PPP1R11, CSNK2B, AXL, TRIM27, ZNF26, TRIM22, MTTP, RLF, CSNK1D, SFPQ, ZNF460, MAT2B, BARD1, RALY, RNASEL, PRPF4B, LMO2, STK38, ATOX1, ZNF451, RBM4, KNTC1, SLC7A8, ADH1A, CRADD, RCBTB2, GHITM, CDKN2A, APOA1, CDKN2C, SLC2A1, SMARCD1, LONRF3, FBXO24, ANKZF1, FBXO22, DDOST, ATG9A, ACTN4, DFFA, STK24, PCTP, TACC3, TMEM106C, NFU1, DCUN1D1, MAD2L1BP, HIF1A, THBD, TIMELESS, HSPB6, FBXO17, IGBP1, PRCP, VAMP5, ZNF587, MFAP4, SPTB, PHKA2, TRAF2, HMGB3, CCDC90B, ADORA2A, ADAMTSL4, ZNF330, TUBB, MEIS2, FBXW4, HIST1H4C, ASF1B, MRC1, TAF6, C14ORF159, SMG5, MEA1, EFEMP1, BRIP1, WHSC1, TEAD3, EHMT2, CDKN1C, EPS15, APEH, ATF5, CDKN1B, CRBN, HDAC1, TRAF3IP3, PTTG1IP, TDG, TBXA2R, ABL1, NOMO2, ZNF410, IER3, E2F3, LEPR, CLSTN1, DPH1, C1ORF35, NDUFAF1, SPRY4, SLC1A5, KIF2C, ALAS1, MAZ, HEY1, CASP1, LOXL2, SUCLA2, CASP2, ANKS1A, MID1IP1, SLC9A3R1, CCNL2, LCK, NCOA6, MAPRE2, ZFPM2, JAM2, CA1, EGLN3, RAG1, CDC73, NFYA, DCN, PIPOX, ANXA6, CHD1L, TSC22D4, TMED1, CD2, GPSM2, CD4, SELPLG, HSPA8, CEBPA, FLT1, GMDS, SNX27, VHL, CEBPG, ANXA1, SAMHD1, DPYSL4, BCS1L, COTL1, UBE2Q1, IFIT1, NEB, HBZ, GDF15, DMBT1, MRPL40, ATP1B1, EFNA1, STAT5A, U2AF2, ZNRF4, RAB1B, PTPN21, CD53, CD2AP, SHH, CD48, KIF13A, HOOK1, DDX11, HOMER3, KIFAP3, RAPGEF6, C7ORF49, RARA, CRY1, BCL7A, GTPBP2, F12, POLH, BYSL, STRN4, RAP1GDS1, RAD9A, TAF6L, HES1, LILRB2, VEGFC, KRT17, BAZ1B, CCR5, ABT1, TXNDC5, VEGFA, EFNA4, ALDOA, FIP1L1, CLCN3, ELL, FCER2, KEL, TAF9B, BANF1, TJAP1, TIPRL, TK1, SLC4A1AP, FBXO3, FBXO9, CDC37L1, KLF5, NOTCH2NL, CDC23, NCKAP1L, CDC20, YTHDC2, RAD54L, EPHA3, CCT7, YY1AP1, TULP3, NUP62, ATP2A2, PTCD3, ETS2, AIRE, POMP, RIT1, CLEC7A, KLF2, NCOR2, PPARD, HIP1R, COPS4, GJA1, GJA4, RBM4B, TNFRSF11A, CEP250, COX17, TOP2A, ICAM1, CCNK, POGZ, CCNH, GZMA, ATP4B, VIL1, FLOT1, POLR1C, GTF2B, RBBP6, CD163, MED4, CKM, G6PD, ADM, SIAH1, REV3L, PRF1, THAP7, PPFIA4, RAP1GAP, SHFM1, LANCL1, ZBTB16, SEC14L4, SFN, CYLD, LPXN, MDC1, NCAPG, FAT4, ETV1, BCL3, CD24, PEX12, BCAS3, GDI1, PLEK, NASP, GMNN, BCKDHB, ATP1A1, ITPR3, HYI, SLC16A3, UBTF, MAPK13, FYN, MAPK14, PHF21A, PGK1, PYGB, CRABP1, TUFT1, CHMP4A, MORF4L2, CBX4, PKMYT1, APOBEC3G, SAE1, ITPKB, TLR6, FLCN, MRPS31, KLHL3, MMP2, PDHB, KLHL2, TTR, PTGIS, TBC1D17, RBCK1, SRRM1, CUTA, DTL, TRIM36, TNS1, TRIM33, RIOK3, FNBP1L, SNRPB, RNF24, SNRPA, SNRPF, DYNLRB1, PRPS2, IFIH1, TSHZ2, FGFR3, SNX2, COPS7A, SOX4, NEDD9, MAP4K1, ENSA, SRF, FXYD6,

					LLGL2, RASAL2, UBE2D3, MTCH1, SCARB1, UBAP2L, NDRG1, ARL6IP5, RNF13, SREBF1, MRPS27, PTPN6, TMEM97, CSTF3, GABARAPL1, MAFB, CPS1, REEP5, CORO1A, C2ORF42, GCM2, PLEKHA5, PHF1, NEDD4, PPID, LAGE3, TROAP, HOXB6, RFX1, KCTD17, SPCS1, CD79A, SCMH1, KCTD13, PLEKHA1, ARHGAP10, TACR3, FOXK2, HPS4, SLA, AKT1, ACVR1B, TDO2, GSN, POU5F1, KLHL22, SHC1, PLCB1, SHC3, PHC2, KHDRBS1, KIAA0907, NOL3, PFKL, RING1, LDB2, MCM3, UBE2B, NLRP1, UBE2O, DDR1, ALDH7A1, CRKL, BTG2, C1QBP, LARP7, CFL1, TNNI3K, KPNA3, PPP1R15A, C1D, CXORF57, HCFC1, TRIB3, NPAS2, ENO2, SLC25A46, SLC39A7, YES1, ENO1, TNPO3, EPO, DCTD, PTPRC, ZBTB7A, IL2RB, NUP153, GIMAP5, MSH5, PSRC1, CEP63, RACGAP1, UBL5, WSB1, TRPC4AP, IGFBP2, IGFBP3, ATP8A1
**GOTERM_CC_DIRECT**	GO:0005829~cytosol	253	0.176782	1.02E-12	LDHA, STAT5A, EHHADH, PPP2R5D, RUSC2, VPS52, MYLIP, SHH, HOOK1, CUL3, CDCA8, HOMER3, KIFAP3, INCENP, CH25H, RPL26L1, RAPGEF6, SMOX, LSM2, LSM1, DDAH2, CRYAB, PCBD1, BYSL, RAP1GDS1, ESPL1, RIC8B, FARP2, GRB10, ALOX15B, CCR2, UBL4A, HARS, MAPK7, MPST, ALDOA, CHKA, NFKBIE, PFKFB3, HSPA1A, NAGK, BANF1, CAPZB, TK1, PPP1R3C, RAC2, RAC1, FBXO3, CDC42EP3, ARHGEF10L, MOCS1, CDC23, UBE2L6, HGD, NCKAP1L, CDC20, GAS2, ARFIP1, DVL1, CCT7, UPF3B, POMP, HGS, TNK1, GRIP1, COPS4, GJA1, DAXX, GTSE1, MSRA, CEP250, SPG21, LMOD1, FMNL1, CDC6, ARHGEF2, POLR1C, GRHPR, RBBP6, MORC2, WAS, KLHDC3, DOK2, CKM, G6PD, OTOF, ADK, SIAH1, TPH1, PPFIA4, RAP1GAP, HK2, DPYS, ZBTB16, SFN, CYLD, SORBS3, CBR1, MTMR1, NCAPG, PDE1A, EXOC4, WIPF2, PPP3CA, EXOC2, RASA1, GDI1, PLEK, GMNN, CSNK2B, TRIM27, TRIM22, PCK1, CSNK1D, MAPK13, FYN, MAPK14, MAT2B, PGK1, DUSP6, RNASEL, ALDH1L1, IL16, CRABP1, ATOX1, CHMP4A, KNTC1, PKMYT1, ADH1B, SAE1, ITPKB, ADH1A, APOBEC3G, ITPKA, KLHL3, KLHL2, CRADD, TBC1D17, CDKN2A, APOA1, CDKN2C, SLC2A1, RGN, RBCK1, SRRM1, ITPK1, SAR1B, STK24, DFFA, NUDT3, PCTP, TAZ, PDE4C, PNPLA4, DDIT4, NFU1, HIF1A, TRIM36, RIOK3, TRIM34, IGBP1, SNRPB, SNRPF, PCCB, SPTB, PHKA2, TRAF2, FGD1, IFIH1, SNX2, RASAL2, UBE2D3, GALE, GLO1, NDRG1, SREBF1, PTPN6, GABARAPL1, SMG5, FN3KRP, EPS15, APEH, PDE7B, CORO1A, CDKN1B, PLEKHA5, HDAC1, NEDD4, FABP4, ABL1, ARHGAP10, IER3, METAP1, DPH1, ARHGAP15, AKT1, KIF2C, CRYL1, TDO2, AKR1C4, GSN, POU5F1, SHC1, CAT, PLCB1, SHC3, CASP1, CASP2, HPD, KHDRBS1, NOL3, PFKL, NUP88, MID1IP1, NLRP1, ARHGAP25, UBE2O, ALDH7A1, CAPN10, CRKL, C1QBP, BTG2, LCK, KPNA3, CA1, PPP1R15A, LCP1, CPLX3, ADH5, EGLN3, ADH6, TRIB3, NPAS2, ENO2, PNPO, YES1, HSPA8, ENO1, DCTD, CENPM, GIMAP4, GMDS, GIMAP6, VHL, SNX27, PSRC1, DPYSL4, CEP63, RACGAP1, UBE2Q1, WSB1, IFIT1, NEB
**GOTERM_CC_DIRECT**	GO:0005737~cytoplasm	341	0.238272	1.25E-08	HMGN1, LDHA, XRCC3, XRCC2, STAT5A, EHHADH, PTPN21, AQP7, CD2AP, HTR1B, DDX11, HOMER3, C7ORF49, ZNF395, RARA, SMOX, DDAH2, LSM1, PDPR, ARC, POLH, CRYAB, MPDZ, PCBD1, BYSL, STRN4, ESPL1, RAD9A, RIC8B, ERMAP, FARP2, HES1, LILRB2, GRB10, KRT17, CCR5, MTF2, ALOX15B, SPAG4, CCR2, VEGFA, HARS, UBL4A, STC1, MAPK7, NEK4, DOC2B, MYBBP1A, SH3GL1, HSD17B10, NFKBIE, ELL, HSPA1A, CAPZB, BANF1, TIPRL, RAC2, RAC1, SLC4A1AP, FBXO3, CDC42EP3, CDC37L1, FBXO9, ARHGEF10L, KLF5, MSTO1, MKI67, NOTCH2NL, CDC20, CD5L, CCT7, SYNE1, YY1AP1, TULP3, UPF3B, NUP62, WDR61, ETS2, POMP, AIRE, HGS, TNK1, CLEC7A, POP7, PLCXD1, BMP6, ELF3, HIP1R, MARCKSL1, UBE2G1, EZH2, BNIP3, RNF187, TTLL7, RASSF7, DAXX, MSRA, ZNF146, SPG21, USP11, LMOD1, USP16, COX17, TOP2A, MAGEH1, CDC6, ARHGEF2, SH3PXD2A, POGZ, LPGAT1, VIL1, TTF1, GRHPR, DECR1, CLIC1, MORC2, TTF2, CARD10, KLHDC3, PJA2, MAST2, G6PD, ADM, PFDN6, ADK, SIAH1, TRIOBP, MKNK2, C11ORF21, LANCL1, SFN, LOC440434,

					LPXN, MTMR1, FAM117A, NCAPG, EXOC4, DHX16, BCL3, PPP3CA, LECT2, BCAS3, RASA1, FLRT3, SSRP1, FLRT2, GDI1, BRD2, SHMT2, NBPF10, PLEK, NASP, GMNN, NBPF11, CSNK2B, TRIM27, PPP1R11, ITPR3, TRIM22, PCK1, CLEC3B, MAPK14, SFPQ, USP48, PYGB, DUSP6, BARD1, ALDH1L1, CRABP1, STK38, IL16, TUFT1, CHMP4A, RBM4, KNTC1, SLC7A8, APOBEC3G, FLCN, CRADD, KLHL2, TTR, CDKN2A, CDKN2C, SLC2A2, RGN, RBMS3, ACTN4, NUDT3, DTL, STK24, DFFA, PNPLA4, TACC3, DDIT4, NFU1, RNF130, MAD2L1BP, TNS1, HIF1A, HSPB6, FNBP1L, TRIM34, IGBP1, CSTB, SNRPA, DYNLRB1, SPTB, TRAF2, FGD1, HMGB3, LST1, SSH3, HDGF, SNX2, TFEB, COPS7A, SOX4, NEDD9, MAP4K1, ENSA, BICC1, SRF, LLGL2, RASAL2, TUBB, SRR, SCARB1, NDRG1, GLO1, UBAP2L, SDF4, IFT140, SREBF1, PTPN6, PHACTR4, TAF6, SMG5, MEA1, MTHFSD, BRIP1, MTRF1L, WHSC1, CDKN1C, EPS15, P2RX5, FBXO38, APEH, ATF5, CORO1A, CDKN1B, CRBN, PLEKHA5, PHF1, HDAC1, THSD1, NEDD4, PPID, TROAP, KCTD17, PTTG1IP, FABP4, ZNF318, CD79A, ABL1, PLEKHA1, CYB5R3, METAP1, E2F3, TACR3, HPS4, NDUFAF1, SPRY4, AKT1, CCHCR1, CRYL1, ALAS1, AKR1C4, HEY1, GSN, POU5F1, NBPF1, KLHL22, PLCB1, PPP1R14B, CASP2, ANKS1A, ZCCHC2, NOL3, PFKL, RING1, UBE2NL, SLC9A3R1, UBE2B, LAP3, UBE2O, LARP6, ALDH7A1, C1QBP, FRMD4A, LARP7, TBCC, CFL1, TNNI3K, ZFPM2, MAPRE2, KPNA3, CA1, PPP1R15A, LCP1, C1D, EGLN3, HCFC1, CDC73, DCN, IVNS1ABP, NPAS2, CHD1L, RGS12, TSC22D4, CYP26B1, PNPO, GPSM2, YES1, ENO1, TNPO3, DCTD, NUP153, MBTPS2, GMDS, SNX27, PSRC1, GRSF1, ANXA1, DPYSL4, COTL1, RACGAP1, UBL5, CYP4A11, IFIT1, GDF15, DMBT1
**GOTERM_CC_DIRECT**	GO:0005654~nucleoplasm	203	0.141845	2.06E-08	HMGN1, XRCC3, XRCC2, STAT5A, U2AF2, PPP2R5D, INTS3, CUL3, CDCA8, DDX11, CLK2, INCENP, INTS7, RARA, LSM2, RPP21, POLH, PCBD1, CRYAB, BYSL, LIG1, PTBP1, RAD9A, HES1, BAZ1B, MTF2, MAPK7, MYBBP1A, PPP2R3B, FIP1L1, ELL, PFKFB3, TAF9B, HSPA1A, BANF1, VPS72, TCF20, SLC4A1AP, FBXO3, KLF5, UBE2L6, CDC23, CDC20, RAD54L, SYNE1, UPF3B, WDR61, ETS2, FNBP4, POMP, H3F3A, CLEC7A, POP7, NCOR2, GTF3A, PPARD, COPS4, EZH2, BNIP3, RNF187, DAXX, GTSE1, MSRA, FANCE, TOP2A, CDC6, CCNK, POGZ, SNAPC1, CCNH, VIL1, TTF1, POLR1C, DECR1, RBBP6, GTF2B, TAF11, MED4, ADK, THOC2, REV3L, SKIV2L, MKNK2, FHL2, SF3B4, SF3B2, MDC1, BCL3, DHX16, PPP3CA, SSRP1, JARID2, GMNN, NASP, PAK1IP1, CSNK2B, TRIM27, ITPR3, TRIM22, CSNK1D, UBTF, SFPQ, MAPK14, PHF21A, USP48, BARD1, DUSP6, PRPF4B, RBM4, MORF4L2, CBX4, PKMYT1, SAE1, PDHB, CDKN2A, RGN, SRRM1, STK24, DTL, DFFA, NFU1, RCL1, HIF1A, TIMELESS, TRIM33, SNRPB, SNRPA, SNRPF, HDGF, SOX4, COPS7A, ENSA, SRF, LLGL2, UBE2D3, HIST1H4C, ASF1B, SREBF1, CSTF3, TAF6, BRIP1, WHSC1, TEAD3, EHMT2, ATF5, PLEKHA5, CDKN1B, PHF1, HDAC1, PPID, RFX1, TDG, ZNF318, SCMH1, ABL1, KCTD13, PLEKHA1, E2F3, FOXK2, DEK, AKT1, ALAS1, HEY1, POU5F1, LOXL2, PHC2, KHDRBS1, ANKS1A, NUP88, RING1, MCM3, MSC, UBE2B, CCNL2, LAP3, LARP7, NCOA6, ZFPM2, KPNA3, C1D, RAG1, EGLN3, TRIB3, HCFC1, CDC73, NFYA, IVNS1ABP, RPA4, NPAS2, CHD1L, SLC39A7, PNPO, HSPA8, TSEN34, FAM50A, NUP153, CENPM, VHL, SNX27, CEBPG, ANXA1, SAMHD1, RACGAP1
**GOTERM_MF_DIRECT**	GO:0019899~enzyme binding	40	0.02795	1.99E-06	TRAF2, ADORA2A, U2AF2, EHHADH, CBX4, HSPA1A, DAXX, BANF1, TSPYL1, AKT1, APOA1, RAC1, RARA, SLC4A2, CD4, CAT, PPP3CA, YES1, PLCB1, TOP2A, CASP2, HSPA8, CUTA, VHL, LDB2, RAD9A, CDC20, COTL1, NLRP1, DVL1, HIF1A, TULP3, ATP2A2, HDAC1, FYN, MAPK14, NCOA6, MAT2B, USP22, SLC27A2
**GOTERM_CC_DIRECT**	GO:0070062~extracellular exosome	194	0.135556	2.33E-06	ATP1B1, LDHA, EFNA1, RUSC2, RAB1B, CD53, KIAA0319L, CD2AP, CUL3, CD48, DDX11, PLOD1, PLOD2, KIFAP3, SERPINE1, RPL26L1, IGLL1, DDAH2, GNG7, ITFG1, F12, CLCA4, PTPRF, PCBD1, CRYAB, PTBP1, RAP1GDS1, F9, TAF6L, PTPRO, PRELP, KRT17, ALOX15B,

					TXNDC5, MPST, ALDOA, FCER2, NAGK, BANF1, CAPZB, RAC2, RAC1, FGL2, SPP2, HGD, NCKAP1L, CD5L, KRT35, CCT7, HYOU1, H3F3A, HGS, MARCKSL1, UBE2G1, COPS4, GJA1, RTN3, MSRA, CEP250, SEMA3G, CETP, VWA1, SCN10A, FMNL1, ICAM1, CAMP, VIL1, FLOT1, CLIC1, DECR1, GRHPR, WAS, G6PD, SLC27A2, C7, GANAB, LANCL1, DPYS, SFN, ITM2C, LOC440434, CBR1, FAT4, SERPINC1, C2, FLRT2, SHMT2, AXL, CSNK2B, ATP1A1, PCK1, TMEM2, CLEC3B, MAPK14, MAT2B, PGK1, PYGB, ALDH1L1, CHMP4A, SLC7A8, CXCL12, PDHB, TTR, APOA1, GHITM, SLC2A1, CUTA, ACTN4, STK24, NUDT3, TRIM36, FNBP1L, PRCP, VAMP5, SNRPB, CSTB, NEU1, MFAP4, PRPS2, SNX2, TUBB, UBE2D3, HIST1H4C, GALE, GLO1, NDRG1, SCARB1, SDF4, ARL6IP5, PTPN6, EFEMP1, REEP5, APEH, OMD, CORO1A, NEDD4, PTTG1IP, FABP4, PLEKHA1, KCTD12, CYB5R3, CLSTN1, CLDN5, IGFBP6, SLC1A5, ACVR1B, CRYL1, AKR1C4, GSN, ACYP1, CAT, SUCLA2, PLCB1, HPD, PFKL, UBE2NL, SLC9A3R1, LAP3, DDR1, ALDH7A1, CRKL, BTG2, CFL1, LCK, CA1, PROS1, LCP1, ADH5, ADH6, ANXA6, ENO2, PNPO, YES1, HSPA8, DPT, ENO1, DCTD, PTPRC, LIPA, GMDS, TMC5, ANXA1, RACGAP1, COTL1, UBL3, HPR, CYP4A11, NEB, HBZ, GDF15, IGFBP2, IGFBP3, DMBT1, ATP8A1
**GOTERM_BP_DIRECT**	GO:0001666~response to hypoxia	25	0.017469	9.71E-06	LDHA, ATP1B1, EGLN3, BNIP3, CXCL12, SRF, MMP2, PLOD1, PLOD2, CAT, CD24, CASP1, LOXL2, ANGPT2, EPO, NOL3, ACTN4, CRYAB, DDIT4, VEGFC, CDKN1B, HIF1A, ADM, CA9, VEGFA
**GOTERM_BP_DIRECT**	GO:0050900~leukocyte migration	20	0.013975	1.69E-05	ICAM1, PTPN6, ATP1B1, SELL, SLC7A8, FPR1, MMP1, SLC16A3, CD48, DOK2, THBD, FYN, LCK, CD2, SHC1, JAM2, YES1, SELPLG, ANGPT2, PROS1
**GOTERM_BP_DIRECT**	GO:0006464~cellular protein modification process	18	0.012577	2.78E-05	PHKA2, STK38, UBE2L6, SAE1, MMP15, TTLL7, UBL5, AKT1, MSRA, UBE2D3, ST3GAL2, PLOD1, PLOD2, UBL4A, ABL1, LOXL2, YES1, FBXO22
**GOTERM_BP_DIRECT**	GO:0006461~protein complex assembly	19	0.013276	2.94E-05	TRAF2, IL2RB, GMNN, VIL1, KNTC1, HFE, NCKAP1L, SLC9A3R1, NDUFAF1, CD2AP, WAS, CARD10, HES1, LPXN, CLGN, KIFAP3, PPID, SLC2A1, KPNA3
**GOTERM_MF_DIRECT**	GO:0042802~identical protein binding	66	0.046117	3.70E-05	LDHA, CHMP4A, CLDN5, BNIP3, KLHL2, HOOK1, AKT1, TTR, TDO2, ALAS1, APOA1, CLK2, SLC2A1, VWA1, CASP2, PHC2, KHDRBS1, PCDHB8, NOL3, PFKL, PCBD1, CRYAB, VIL1, WAS, INHBA, G6PD, ZNF783, LCK, VEGFA, SNRPA, SIAH1, MAPRE2, LCP1, PRPS2, MPST, SH3GL1, ALDOA, TRAF2, THAP7, ERBB3, ADORA2A, FHL2, HCFC1, SFN, ZBTB16, TK1, GPSM2, GALE, LECT2, SDF4, TNPO3, DCTD, SHMT2, NUP153, GMDS, TRIM27, CSNK2B, HGD, DVL1, EPS15, CCT7, APEH, FYN, KCTD17, AIRE, KCTD13
**GOTERM_CC_DIRECT**	GO:0016020~membrane	151	0.10551	5.74E-05	KIFC1, ATP1B1, LDHA, EFNA1, TNFSF15, VPS52, SLC7A8, PKMYT1, ITPKB, CD48, CUL3, GLT8D1, SLC2A6, SLC2A2, SLC2A1, DHX34, IGLL1, SLC4A2, ANKZF1, DDOST, CUTA, ATG9A, STK24, BYSL, PTBP1, STRN4, PNPLA4, MARK2, OGFRL1, TMEM106C, VEGFC, LILRB2, ALOX15B, VEGFA, UBL4A, NEU1, DOC2B, MYBBP1A, DYNLRB1, ALDOA, CLCN3, KIAA1109, LST1, ADORA2A, MMD, LRBA, SNX2, MAP4K1, SFXN1, CAPZB, SLCO2A1, SLC29A1, RAC2, FOLR2, MTCH1, RAC1, HIST1H4C, ARL6IP5, SDF4, GAL3ST1, CSF1R, PTPN6, MKI67, NCKAP1L, GAS2, CD5L, EPS15, HYOU1, CORO1A, CRBN, PLEKHA5, ATP2A2, PTTG1IP, RIT1, TNK1, NCOR2, CYB5R3, SLC20A1, LEPR, HPS4, CYC1, MAN1B1, ALG5, NUP188, ARHGAP15, GTSE1, SPRY4, SLC1A5, ACVR1B, KIF2C, MSRA, SLC1A3, PIGG, LMOD1, CAT, LOXL2, CASP2, SLC22A1, FMNL1, KHDRBS1, ICAM1, NOL3, LPGAT1, PFKL, FLOT1, CLIC1, MCM3, SLC9A3R1, CD163, DDR1, SLC26A6, MED4, G6PD, C1QBP, CFL1, PPP1R15A, DEGS1, PRF1, GANAB, RAP1GAP, TBC1D8, HK2, HCFC1, ANXA6, LPXN, NCAPG, FAT4, EXOC4, SLC39A7,

					CD4, PPP3CA, CD24, SELPLG, EXOC2, HSPA8, SLC39A1, ENO1, PTPRC, IL2RB, PLEK, VHL, RRBP1, SELL, TRIM27, ATP1A1, ITPR3, SLC16A3, PGK1, DMBT1, ATP8A1, PYGB
**GOTERM_BP_DIRECT**	GO:0090023~positive regulation of neutrophil chemotaxis	8	0.00559	8.59E-05	CCR7, IL23A, C1QBP, RAC2, CCL21, RAC1, NCKAP1L, CCL19
**GOTERM_BP_DIRECT**	GO:0007165~signal transduction	90	0.062887	1.30E-04	SLC20A1, CRABP1, PPP2R5D, IGFBP6, TNFSF15, GJA1, ITPKB, DEK, CD53, RASSF7, TLR6, ARHGAP15, CXCL12, CD2AP, ITPKA, CRADD, CD48, AKT1, ACVR1B, TNFRSF11A, EVI2A, KIFAP3, IL4R, SHC1, RARA, CASP1, PLCB1, SHC3, ITPK1, ANGPT2, KHDRBS1, DFFA, STK24, PDE4C, CLIC1, ARHGAP25, VEGFC, LILRB2, GRB10, DOK2, HIF1A, THBD, KRT17, ADM, IGBP1, MAPRE2, MAPK7, CLEC5A, SH3GL1, TRAF2, FGD1, CHKA, RAP1GAP, ERBB3, HDGF, FPR1, NEDD9, SP110, SFN, OR1G1, RASAL2, LPXN, RGS12, RAC2, PDE1A, TMED1, CD4, CDC42EP3, RASA1, CSF1R, EPO, CREBL2, MRC1, GDI1, GABRE, IL2RB, SNX27, CSNK2B, AXL, ANXA1, RACGAP1, P2RX5, PDE7B, CSNK1D, MAPK14, HGS, RIT1, IGFBP2, GDF15, ARHGAP10
**GOTERM_MF_DIRECT**	GO:0008134~transcription factor binding	31	0.021661	1.96E-04	PPARD, TAF9B, FHL2, DAXX, SRF, SORBS3, MEIS2, CDKN2A, HEY1, POU5F1, BCL3, RARA, BCAS3, CRY1, CEBPA, KLF5, ARHGEF2, VHL, MAFB, CEBPG, CSNK2B, GTF2B, HES1, HIF1A, HDAC1, C1QBP, PPID, TDG, ZFPM2, MYBBP1A, NCOR2
**GOTERM_MF_DIRECT**	GO:0004842~ubiquitin-protein transferase activity	34	0.023757	2.53E-04	TRAF2, UBE2G1, RAG1, RNF187, MYLIP, KLHL3, KLHL2, CUL3, UBE2D3, KLHL22, RBCK1, FBXO24, FBXO3, PEX12, FBXO9, FBXO22, RNF13, DTL, VHL, TRIM27, UBE2L6, CDC23, RBBP6, UBE2B, WSB1, PJA2, UBE2O, RNF130, TRIM36, TRIM33, NEDD4, SIAH1, KCTD13, BARD1
**GOTERM_CC_DIRECT**	GO:0015629~actin cytoskeleton	25	0.017469	2.79E-04	ALDOA, TRIOBP, ARC, ACTN4, ELL, KNTC1, FHL2, IVNS1ABP, SLC9A3R1, WAS, CAPZB, CD2AP, KLHL2, SLC16A3, MSRA, CORO1A, NEB, NCAPG, GSN, CFL1, RARA, ABL1, CDC42EP3, LCP1, SPTB
**GOTERM_BP_DIRECT**	GO:0045638~negative regulation of myeloid cell differentiation	7	0.004891	2.94E-04	MEIS2, HMGB3, WDR61, GPR171, CDC73, ITPKB, ZBTB16
**GOTERM_BP_DIRECT**	GO:0016925~protein sumoylation	17	0.011879	3.61E-04	NUP153, IFIH1, NUP88, ZNF451, CBX4, RING1, SAE1, NUP188, CDCA8, CDKN2A, NUP62, MDC1, INCENP, TDG, SCMH1, TOP2A, PHC2
**GOTERM_BP_DIRECT**	GO:0006513~protein monoubiquitination	8	0.00559	4.54E-04	CUL3, UBE2O, UBE2D3, NEDD4, DTL, KLHL22, PEX12, UBE2B
**GOTERM_BP_DIRECT**	GO:0031529~ruffle organization	6	0.004192	5.07E-04	CCR7, PLEK, CCL21, RAC1, CSF1R, PLEKHA1
**GOTERM_BP_DIRECT**	GO:0006974~cellular response to DNA damage stimulus	24	0.01677	5.27E-04	CCNK, XRCC3, DTL, EGLN3, RAD9A, INTS3, RBBP6, UBE2B, AKT1, NPAS2, CHD1L, TIMELESS, BAZ1B, PHF1, BTG2, DDX11, NCOA6, BCL3, NEK4, ABL1, USP16, TOP2A, PPP1R15A, BARD1
**GOTERM_MF_DIRECT**	GO:0008022~protein C-terminus binding	22	0.015372	5.44E-04	ATP1B1, MKI67, GRIP1, MPDZ, SAE1, CDC20, MID1IP1, ZBTB16, BANF1, CD2AP, CORO1A, CEP250, ATP2A2, MDC1, HOMER3, LCK, TNNI3K, SIAH1, PEX12, ABL1, KPNA3, TOP2A
**GOTERM_MF_DIRECT**	GO:0016597~amino acid binding	7	0.004891	7.21E-04	SHMT2, TDO2, SLC1A3, OTC, DPYS, TPH1, DDAH2
**GOTERM_MF_DIRECT**	GO:0042826~histone deacetylase binding	15	0.010481	8.25E-04	SMG5, GMNN, CDC20, HSPA1A, RAD9A, SRF, HES1, HIF1A, HDAC1, SFPQ, RAC1, RARA, CRY1, TOP2A, NCOR2
**GOTERM_BP_DIRECT**	GO:0008360~regulation of cell shape	18	0.012577	9.64E-04	FMNL1, ALDOA, ICAM1, FGD1, LST1, VIL1, ANXA1, GAS2, SLC9A3R1, ARHGAP15, CORO1A, CSNK1D, FYN, VEGFA, WIPF2, CDC42EP3, RASA1, CSF1R
**GOTERM_BP_DIRECT**	GO:0051289~protein homotetramerization	11	0.007686	9.68E-04	ALDOA, SHMT2, PFKL, PCBD1, SRR, SAMHD1, DPYS, CAT, DECR1, USP16, TK1

**GOTERM_CC_DIRECT**	GO:0005884~actin filament	11	0.007686	0.00145379	CORO1A, RAC2, FYN, RAC1, WIPF2, GAS2, LMOD1, YES1, CAPZB, LCP1, MARK2
**GOTERM_BP_DIRECT**	GO:0006469~negative regulation of protein kinase activity	14	0.009782	0.0018197	FLRT3, PTPRC, FLRT2, ADORA2A, TRIM27, TRIB3, SFN, DCN, CAMK2N1, DVL1, AKT1, CDKN2A, FABP4, RGN
**GOTERM_BP_DIRECT**	GO:0061621~canonical glycolysis	7	0.004891	0.00182358	ALDOA, PFKL, PFKFB3, ENO2, HK2, PGK1, ENO1
**GOTERM_CC_DIRECT**	GO:0043234~protein complex	36	0.025155	0.00185196	SNX2, VPS72, CD48, AKT1, TTR, CDCA8, TUBB, CDKN2A, CEP250, INCENP, BCL3, HIST1H4C, ASF1B, TOP2A, DDOST, HIGD1A, SREBF1, GDI1, ARHGEF2, ACTN4, NASP, STRN4, ANXA1, CDC20, ATP1A1, CPS1, GNAT3, CORO1A, CDKN1B, ATP2A2, HDAC1, NEB, H3F3A, PRPS2, SLC27A5, SPTB
**GOTERM_CC_DIRECT**	GO:0005634~nucleus	314	0.219406	0.00194505	MRPL40, HMGN1, KIFC1, LDHA, XRCC3, STAT5A, RUSC1, U2AF2, PPP2R5D, INTS3, DNASE1L3, CUL3, CDCA8, DDX11, CLK2, ZNF248, DHX34, C7ORF49, ZNF395, RARA, SMOX, LSM1, CRY1, CRYAB, PMCH, LIG1, BYSL, PTBP1, ESPL1, RAD9A, TAF6L, HES1, ZNF787, SPAG7, ABT1, ZNF783, MTF2, ALOX15B, UBL4A, STC1, MAPK7, MYBBP1A, ZNF613, ALDOA, PPP2R3B, KIAA1109, PUS1, ELL, BANF1, VPS72, TCF20, ZNF225, RAC1, ZNF222, SLC4A1AP, MOCS1, MKI67, CDC20, YTHDC2, RAD54L, SYNE1, YY1AP1, TULP3, UPF3B, WDR61, ETS2, POMP, AIRE, H3F3A, KLF2, RERE, NCOR2, POP7, GTF3A, HLF, PPARD, ELF3, PCDHA2, ZNF532, COPS4, EZH2, BNIP3, RNF187, DGCR14, DAXX, MBTD1, ZNF146, USP11, FANCE, USP16, TOP2A, ANGPT2, CDC6, CCNK, POGZ, CCNH, SNAPC1, GZMA, TTF1, DECR1, CLIC1, RBBP6, MORC2, GTF2B, MED4, G6PD, ZNF692, ADK, SIAH1, REV3L, TRIOBP, SKIV2L, MKNK2, FHL2, ZBTB16, SFN, SORBS3, LPXN, MDC1, NCAPG, PDE1A, DHX16, BCL3, ETV1, MEIS3P1, PPP3CA, BCAS3, SSRP1, BRD2, SHMT2, BRD3, JARID2, NASP, GMNN, CSNK2B, TRIM27, PPP1R11, ZNF26, TRIM22, RLF, CSNK1D, UBTF, FYN, MAPK14, SFPQ, ZNF460, MAT2B, REPIN1, BARD1, RALY, LMO2, STK38, IL16, CHMP4A, MORF4L2, RBM4, ZNF451, KNTC1, CBX4, SAE1, ITPKB, FLCN, MMP2, PDHB, CRADD, APOA1, CDKN2A, PTGIS, CDKN2C, RGN, SRRM1, FBXO22, ACTN4, NUDT3, DTL, STK24, DFFA, TAZ, RNF130, NFU1, DCUN1D1, MAD2L1BP, HIF1A, TRIM33, HSPB6, TIMELESS, SNRPB, ZNF587, TSHZ2, IFIH1, FGFR3, HMGB3, SSH3, TFEB, SOX4, NEDD9, SP110, SRF, ZNF330, TUBB, MEIS2, NDRG1, HIST1H4C, ASF1B, STK19, CREBL2, SREBF1, PTPN6, CSTF3, TAF6, SMG5, MAFB, BRIP1, WHSC1, TEAD3, EHMT2, CDKN1C, FBXO38, ATF5, CORO1A, GCM2, C2ORF42, CDKN1B, CRBN, PHF1, HDAC1, NEDD4, PPID, LAGE3, HOXB6, RFX1, TDG, PTTG1IP, FABP4, ABL1, SCMH1, ZNF410, IER3, METAP1, E2F3, TACR3, CYC1, CLSTN1, FOXK2, DPH1, DEK, AKT1, CCHCR1, KIF2C, CRYL1, MAZ, HEY1, GSN, POU5F1, S100PBP, PLCB1, LOXL2, CASP2, PHC2, KHDRBS1, KIAA0907, RING1, LDB2, UBE2NL, MID1IP1, MCM3, SLC9A3R1, UBE2B, MSC, NLRP1, CCNL2, LAP3, UBE2O, LARP6, ALDH7A1, C1QBP, LARP7, CFL1, NCOA6, TNNI3K, ZFPM2, KPNA3, C1D, TRA2A, RAG1, EGLN3, HCFC1, TRIB3, CDC73, NFYA, RPA4, TSPYL1, NPAS2, FAM107A, CHD1L, RGS12, TSC22D4, HSPA8, TSEN34, ENO1, FAM50A, CEBPA, ZBTB7A, VHL, CEBPG, ANXA1, SAMHD1, COTL1, RACGAP1, UBE2Q1, UBL5, F8A1, GDF15, IGFBP3
**GOTERM_BP_DIRECT**	GO:0042493~response to drug	29	0.020264	0.00247375	LDHA, ADORA2A, HTR1B, SLC1A3, APOA1, SRR, CAT, HTR1F, SREBF1, ICAM1, RPP21, OTC, ANXA1, NCKAP1L, ATP1A1, CPS1, RAD54L, UBE2B, INHBA, VEGFC, CDKN1B, HDAC1, CA9, FYN, LCK, TBXA2R, ABL1, IGFBP2, DUSP6
**GOTERM_BP_DIRECT**	GO:2000107~negative regulation of leukocyte apoptotic process	4	0.002795	0.00248796	CCR7, CCL21, CCL19, CXCL12
**GOTERM_BP_DIRECT**	GO:0001768~establishment of T cell polarity	4	0.002795	0.00248796	CCR7, CCL21, CYP26B1, CCL19

**GOTERM_BP_DIRECT**	GO:0070534~protein K63-linked ubiquitination	8	0.00559	0.00262429	TRAF2, UBE2O, CDKN2A, NEDD4, UBE2G1, TRIM27, UBE2NL, UBE2B
**GOTERM_BP_DIRECT**	GO:0061418~regulation of transcription from RNA polymerase II promoter in response to hypoxia	7	0.004891	0.00273085	UBE2D3, HIF1A, CA9, VHL, VEGFA, EGLN3, EPO
**GOTERM_BP_DIRECT**	GO:0046718~viral entry into host cell	12	0.008385	0.00276396	EPS15, MRC1, ICAM1, SLC1A5, NUP153, AXL, SCARB1, HSPA1A, KPNA3, CLEC5A, SELPLG, SLC10A1
**GOTERM_BP_DIRECT**	GO:0042326~negative regulation of phosphorylation	6	0.004192	0.00302689	CDKN1C, INHBA, GRB10, CDKN2A, CDKN1B, CDKN2C
**GOTERM_BP_DIRECT**	GO:0044267~cellular protein metabolic process	15	0.010481	0.00324631	ADORA2A, IGFBP6, UBE2L6, TTF1, MMP2, MMP1, TTR, APOA1, GSN, H3F3A, HIST1H4C, SIAH1, IGFBP2, IGFBP3, DMBT1
**GOTERM_MF_DIRECT**	GO:0001618~virus receptor activity	11	0.007686	0.00328402	MRC1, ICAM1, SLC1A5, CCR5, AXL, SCARB1, CD4, HSPA1A, CLEC5A, SELPLG, SLC10A1
**GOTERM_MF_DIRECT**	GO:0019888~protein phosphatase regulator activity	7	0.004891	0.00335774	PPP2R3B, PPP1R3C, PPME1, PPP2R5D, IGBP1, ENSA, PPP1R15A
**GOTERM_BP_DIRECT**	GO:0006919~activation of cysteine-type endopeptidase activity involved in apoptotic process	12	0.008385	0.00369932	TRAF2, CDKN2A, CDKN1B, HIP1R, MTCH1, LCK, TNFSF15, EGLN3, CASP1, CASP2, NLRP1, CRADD
**GOTERM_BP_DIRECT**	GO:0000077~DNA damage checkpoint	7	0.004891	0.00393967	MAPK14, INTS7, BRIP1, RAD9A, CEP63, RPA4, TIPRL
**GOTERM_MF_DIRECT**	GO:0005524~ATP binding	102	0.071272	0.0040464	KIFC1, ATP1B1, XRCC3, RNASEL, PRPF4B, XRCC2, ADCY7, STK38, PKMYT1, ITPKB, ITPKA, KIF13A, DDX11, CLK2, MAP3K9, DHX34, ITPK1, STK24, LIG1, ABCC10, MARK2, BAZ1B, RIOK3, HARS, MAPK7, NEK4, PCCB, PRPS2, CHKA, ABCA7, IFIH1, CLCN3, FGFR3, PFKFB3, ERBB3, MAP4K1, HSPA1A, NAGK, TK1, UBE2D3, SRR, STK19, CSF1R, MKI67, BRIP1, UBE2L6, YTHDC2, CPS1, RAD54L, EPHA3, CCT7, P2RX5, HYOU1, ATP2A2, KCNJ8, ATP2A3, TNK1, ABL1, UBE2G1, TTLL7, AKT1, ACVR1B, KIF2C, SUCLA2, TOP2A, ATP8B4, CDC6, PFKL, MCM3, UBE2B, NLRP1, TTF2, DDR1, UBE2O, MAST2, CKM, ADK, LCK, TNNI3K, SLC27A2, SLC27A5, SKIV2L, MKNK2, HK2, TRIB3, ITM2C, CHD1L, DHX16, YES1, HSPA8, FLT1, MSH5, AXL, BCS1L, ATP1A1, UBE2Q1, CSNK1D, FYN, MAPK13, MAPK14, PGK1, ATP8A1
**GOTERM_BP_DIRECT**	GO:0000122~negative regulation of transcription from RNA polymerase II promoter	55	0.038431	0.00445331	PPARD, EFNA1, EZH2, CBX4, FLCN, SHH, CUL3, HEY1, POU5F1, RARA, CRY1, MSC, HES1, TIMELESS, TRIM33, BTG2, C1QBP, MTF2, IGBP1, VEGFA, ZFPM2, ZNF613, TSHZ2, TAF9B, HDGF, FHL2, TRIB3, HCFC1, CDC73, ZBTB16, VPS72, SORBS3, UBE2D3, MEIS2, EPO, KLF5, SREBF1, CEBPA, ZBTB7A, JARID2, VHL, TRIM27, WHSC1, EHMT2, CDKN1C, HDAC1, SFPQ, PPID, ETS2, HBZ, TDG, PHF21A, NCOR2, RERE, BMP6
**GOTERM_MF_DIRECT**	GO:0003682~chromatin binding	34	0.023757	0.00457878	HMGN1, TSHZ2, EZH2, CBX4, HCFC1, DEK, DDX11, SMARCD1, BCAS3, LOXL2, TOP2A, SREBF1, SSRP1, SHMT2, BRD2, BRD3, JARID2, CSNK2B, RING1, TTF1, WHSC1, KLHDC3, ATF5, BAZ1B, HDAC1, UBTF, PHF1, NUP62, SFPQ, NCOA6, AIRE, PHF21A, RERE, NCOR2
**GOTERM_BP_DIRECT**	GO:0050821~protein stabilization	16	0.01118	0.00474973	ATP1B1, VHL, HIP1R, HPS4, TAF9B, HCFC1, SOX4, HSPA1A, UBE2B, GTSE1, DVL1, CCT7, APOA1, CDKN2A, CDC37L1, CREBL2
**GOTERM_BP_DIRECT**	GO:0051209~release of sequestered calcium ion into cytosol	8	0.00559	0.00478655	PTPRC, HTR1B, CCR7, CCL21, LCK, CCL19, ITPR3, HTR1F
**GOTERM_BP_DIRECT**	GO:0008285~negative regulation of cell proliferation	34	0.023757	0.00525086	ADORA2A, IGFBP6, SOX4, CDC73, ZBTB16, SRF, CDKN2A, CDKN2C, KIFAP3, NDRG1, RARA, DPT, CSF1R, CEBPA, CDC6, PTPN6, TAF6, JARID2, VHL, CSNK2B, BRIP1, SLC9A3R1, ATF5, DDR1, VEGFC, INHBA, CDKN1B, NUP62, BTG2, ADM, HDAC1, ALOX15B, HGS, IGFBP3
**GOTERM_BP_DIRECT**	GO:0002548~monocyte chemotaxis	8	0.00559	0.0054917	TNFRSF11A, FLT1, FOLR2, CCL21, ANXA1, CCL19, CCL16, PTPRO
**GOTERM_BP_DIRECT**	GO:0007595~lactation	8	0.00559	0.0054917	SLC29A1, DDR1, HIF1A, STAT5A, VEGFA, HK2, SERPINC1, NCOR2
**GOTERM_BP_DIRECT**	GO:0007566~embryo implantation	8	0.00559	0.0054917	DDR1, PPARD, PTGIS, H3F3A, STC1, UBE2Q1, MMP2, EPO

**GOTERM_BP_DIRECT**	GO:0006369~termination of RNA polymerase II transcription	10	0.006987	0.00560942	FIP1L1, CSTF3, MAZ, UPF3B, U2AF2, SNRPB, SRRM1, THOC2, SNRPF, TTF2
**GOTERM_MF_DIRECT**	GO:0005102~receptor binding	31	0.021661	0.00604068	ACOX2, NXPH4, EFNA1, EHHADH, IGFBP6, TNFSF15, GJA1, HFE, HSPA1A, ENSA, CXCL12, PIPOX, TMED1, SERPINE1, SMARCD1, CD2, RARA, CAT, YES1, ANGPT2, SELPLG, RASA1, SLC39A1, ANXA1, CSNK2B, SLC9A3R1, ADM, TNK1, ABL1, IGFBP2, SLC27A2
**GOTERM_BP_DIRECT**	GO:0048538~thymus development	8	0.00559	0.00627184	HES1, CRKL, JARID2, MAFB, RAG1, ABL1, SRF, SHH
**GOTERM_BP_DIRECT**	GO:0030850~prostate gland development	4	0.002795	0.0064402	ALOX15B, ANXA1, RARA, SHH
**GOTERM_BP_DIRECT**	GO:2001020~regulation of response to DNA damage stimulus	4	0.002795	0.0064402	IER3, NPAS2, NEK4, ABL1
**GOTERM_BP_DIRECT**	GO:0045737~positive regulation of cyclin-dependent protein serine/threonine kinase activity	6	0.004192	0.00698531	CDC6, CCNK, CDKN1B, CCNH, PSRC1, CCNL2
**GOTERM_BP_DIRECT**	GO:0045892~negative regulation of transcription, DNA-templated	40	0.02795	0.00730767	THAP7, PPARD, ELF3, EZH2, CBX4, FHL2, TRIB3, ZBTB16, DAXX, CDKN2A, HEY1, TSC22D4, BCL3, RARA, BCL7A, LOXL2, PLCB1, CRY1, HSPA8, ENO1, KHDRBS1, CEBPA, ZBTB7A, JARID2, GMNN, RING1, CDKN1C, HES1, ATF5, YY1AP1, CDKN1B, TIMELESS, HDAC1, TRIM33, SFPQ, FABP4, ZFPM2, SCMH1, MYBBP1A, C1D
**GOTERM_BP_DIRECT**	GO:0006096~glycolytic process	7	0.004891	0.00748113	ALDOA, LDHA, PFKL, ENO2, HK2, PGK1, ENO1
**GOTERM_BP_DIRECT**	GO:0043065~positive regulation of apoptotic process	27	0.018866	0.00761465	FGD1, LDHA, HIP1R, ADAMTSL4, BNIP3, SOX4, ZBTB16, FLCN, AKT1, CDKN2A, MAP3K9, MTCH1, RAC1, CASP2, ARL6IP5, TOP2A, ARHGEF2, DFFA, GZMA, ADM, C1QBP, PPID, SIAH1, ABL1, IGFBP3, DUSP6, BARD1
**GOTERM_BP_DIRECT**	GO:0008203~cholesterol metabolic process	10	0.006987	0.00835315	CEBPA, SREBF1, PPARD, APOA1, MBTPS2, LEPR, CH25H, CETP, CAT, LIPC
**GOTERM_BP_DIRECT**	GO:0006915~apoptotic process	44	0.030745	0.00853161	IER3, PACS2, PRF1, PPARD, HIP1R, ADORA2A, LY86, ADAMTSL4, GPR65, EGLN3, GJA1, BNIP3, ZBTB16, RASSF7, DAXX, RTN3, UBE2D3, GHITM, CDKN2A, MAP3K9, MTCH1, CD2, CASP1, CASP2, MAGEH1, EBAG9, EPO, PTPN6, DFFA, GZMA, VIL1, ESPL1, GAS2, CD5L, NLRP1, RNF130, C1QBP, ALOX15B, PPID, MAPK14, SIAH1, IGFBP3, PPP1R15A, C1D
**GOTERM_CC_DIRECT**	GO:0005925~focal adhesion	32	0.02236	0.00877184	TRIOBP, FGFR3, FHL2, NEDD9, GJA1, HSPA1A, SPRY4, ANXA6, LPXN, SORBS3, RAC2, GSN, MDC1, RAC1, SLC4A2, CAT, YES1, HSPA8, FLRT3, ICAM1, PTPRC, FLRT2, ARHGEF2, FLT1, ACTN4, FLOT1, ANXA1, LAP3, HYOU1, TNS1, CFL1, LCP1
**GOTERM_BP_DIRECT**	GO:0070301~cellular response to hydrogen peroxide	9	0.006289	0.00890559	IL18RAP, EZH2, ANXA1, AXL, BNIP3, MAPK7, ABL1, KLF2, PLEKHA1
**GOTERM_MF_DIRECT**	GO:0042803~protein homodimerization activity	54	0.037732	0.00928428	HIP1R, CHMP4A, HPS4, BNIP3, APOBEC3G, DAXX, CUL3, CRYL1, PLOD1, MAP3K9, CAT, PLCB1, TOP2A, SLC22A1, ACTN4, GZMA, CRYAB, VIL1, GRHPR, PTPRO, HES1, INHBB, G6PD, TIMELESS, HSPB6, CCR2, VEGFA, PRPS2, CHKA, CLCN3, RAP1GAP, ERBB3, ADH5, RAG1, SNX2, ZBTB16, BANF1, SRF, ANXA6, MTMR1, CD2, SRR, CD4, SCARB1, GALE, CSF1R, FLRT3, CEBPA, PLEK, ANXA1, SYNE1, CORO1A, TDG, BARD1
**GOTERM_MF_DIRECT**	GO:0044822~poly(A) RNA binding	78	0.054502	0.00929732	MRPL40, RALY, PRPF4B, U2AF2, RBM4, DEK, PDIA4, C1ORF35, MRPS31, RBM4B, MAZ, CDKN2A, POU5F1, DHX34, SRRM1, LSM2, LSM1, TOP2A, FNDC3B, KHDRBS1, MRPL2, ACTN4, BYSL, PTBP1, RBBP6, MARK2, UBE2O, TNS1, CRKL, ABT1, LARP7, ADK, CSTB, SNRPB, SNRPA, MYBBP1A, ALDOA, HSD17B10, FIP1L1, CXORF57, GANAB, HMGB3, PUS1, TRA2A, HDGF, BICC1, DCN, SF3B4, SF3B2, TCF20,

					DHX16, HIST1H4C, UBAP2L, HSPA8, ENO1, FAM50A, SSRP1, CSTF3, MRPS28, NBPF10, MKI67, RRBP1, GRSF1, MTHFSD, YTHDC2, SLC16A3, APEH, CORO1A, MRPL22, SYNE1, UPF3B, UBTF, PTCD3, SFPQ, HOXB6, POP7, KCTD12, REPIN1
**GOTERM_BP_DIRECT**	GO:1901741~positive regulation of myoblast fusion	5	0.003494	0.01002924	IL4R, MAPK14, FLOT1, CD53, GDF15
**GOTERM_BP_DIRECT**	GO:0042789~mRNA transcription from RNA polymerase II promoter	5	0.003494	0.01002924	SREBF1, HIF1A, LMO2, POU5F1, SRF
**GOTERM_BP_DIRECT**	GO:0002407~dendritic cell chemotaxis	5	0.003494	0.01002924	CCR7, CCR5, CCL21, CCR2, CCL19
**GOTERM_MF_DIRECT**	GO:0051020~GTPase binding	6	0.004192	0.0100796	FNBP1L, BNIP3, RASA1, LCP1, SH3GL1, ENO1
**GOTERM_BP_DIRECT**	GO:0042110~T cell activation	8	0.00559	0.01023512	CD48, NEDD4, FYN, CD2, PPP3CA, CLEC7A, DDOST, WAS
**GOTERM_BP_DIRECT**	GO:0031100~organ regeneration	8	0.00559	0.01023512	APOA1, ADM, MKI67, AXL, ANGPT2, CXCL12, PRPS2, LCP1
**GOTERM_BP_DIRECT**	GO:0071456~cellular response to hypoxia	12	0.008385	0.0110152	SLC29A1, AKT1, ICAM1, HYOU1, PPARD, HIF1A, PTGIS, VEGFA, BNIP3, STC1, NDRG1, PCK1
**GOTERM_BP_DIRECT**	GO:0007080~mitotic metaphase plate congression	7	0.004891	0.01133659	CUL3, KIFC1, KIF2C, CDCA8, CHMP4A, PSRC1, CDC23
**GOTERM_BP_DIRECT**	GO:0010862~positive regulation of pathway-restricted SMAD protein phosphorylation	8	0.00559	0.01145966	INHBB, ACVR1B, INHBA, INHBE, CSNK2B, HFE, GDF15, BMP6
**GOTERM_CC_DIRECT**	GO:0031464~Cul4A-RING E3 ubiquitin ligase complex	4	0.002795	0.01174741	CRBN, CDKN1B, DTL, TRPC4AP
**GOTERM_MF_DIRECT**	GO:0051721~protein phosphatase 2A binding	6	0.004192	0.01185066	AKT1, PPME1, SMG5, IGBP1, STRN4, ENSA
**GOTERM_BP_DIRECT**	GO:0042102~positive regulation of T cell proliferation	9	0.006289	0.01203866	HES1, PTPRC, LILRB2, CORO1A, IL23A, ANXA1, NCKAP1L, CCL19, CD4
**GOTERM_BP_DIRECT**	GO:0042267~natural killer cell mediated cytotoxicity	5	0.003494	0.01237363	PTPN6, TUBB, MICB, CEBPG, SLAMF7
**GOTERM_BP_DIRECT**	GO:0001771~immunological synapse formation	4	0.002795	0.0127639	PRF1, CORO1A, CCL21, CCL19
**GOTERM_BP_DIRECT**	GO:0008380~RNA splicing	17	0.011879	0.01283801	NOL3, PRPF4B, PTBP1, RBM4, IVNS1ABP, SF3B4, TTF2, SF3B2, RBM4B, C1QBP, SFPQ, SNRPB, SRRM1, DHX16, SNRPF, THOC2, LSM1
**GOTERM_BP_DIRECT**	GO:0043066~negative regulation of apoptotic process	36	0.025155	0.01340703	IER3, PPARD, TAF9B, CBX4, FHL2, BNIP3, SHH, AKT1, KIFAP3, BCL3, RGN, GLO1, RARA, CAT, DDAH2, HIGD1A, CSF1R, IL2RB, NOL3, VHL, CRYAB, PRG1, ANXA1, NCKAP1L, UBE2B, ATF5, CDKN1B, NUP62, HDAC1, TXNDC5, CFL1, VEGFA, MAPK7, CLEC5A, ARHGAP10, BARD1
**GOTERM_CC_DIRECT**	GO:0001891~phagocytic cup	5	0.003494	0.01357143	ABCA7, CORO1A, RAC1, ANXA1, LCP1
**GOTERM_BP_DIRECT**	GO:0071222~cellular response to lipopolysaccharide	13	0.009084	0.01444348	MRC1, ICAM1, PPARD, CAMP, AXL, CDC73, LILRB2, CCR5, MAPK14, SERPINE1, TBXA2R, RARA, ABL1
**GOTERM_BP_DIRECT**	GO:0016569~covalent chromatin modification	13	0.009084	0.01444348	BRD2, MBTD1, BRD3, PHF1, JARID2, MTF2, SMARCD1, TDG, CBX4, DEK, ASF1B, DAXX, VPS72
**GOTERM_BP_DIRECT**	GO:0000079~regulation of cyclin-dependent protein serine/threonine kinase activity	7	0.004891	0.01457704	CDC6, CCNK, CDKN1B, CDKN2C, PKMYT1, SFN, CCNL2
**GOTERM_BP_DIRECT**	GO:0043536~positive regulation of blood vessel endothelial cell migration	5	0.003494	0.01504115	AKT1, VEGFC, MAPK14, STAT5A, VEGFA
**GOTERM_BP_DIRECT**	GO:0051882~mitochondrial depolarization	3	0.002096	0.01510083	CDKN2A, ABL1, CASP1

**GOTERM_MF_DIRECT**	GO:0043548~phosphatidylinositol 3-kinase binding	5	0.003494	0.01523732	CORO1A, FYN, LCK, AXL, ATP1A1
**GOTERM_BP_DIRECT**	GO:0034976~response to endoplasmic reticulum stress	10	0.006987	0.01545759	HYOU1, TRAF2, MBTPS2, ATP2A2, TXNDC5, FLOT1, TRIB3, PDIA4, ABL1, PPP1R15A
**GOTERM_BP_DIRECT**	GO:0000082~G1/S transition of mitotic cell cycle	12	0.008385	0.01684198	CUL3, ACVR1B, INHBA, CDC6, CDKN2A, CDKN1B, CDKN2C, CCNH, PKMYT1, PPP3CA, MCM3, RPA4
**GOTERM_BP_DIRECT**	GO:0043249~erythrocyte maturation	4	0.002795	0.01688127	G6PD, HBZ, KLF2, EPO
**GOTERM_MF_DIRECT**	GO:0004861~cyclin-dependent protein serine/threonine kinase inhibitor activity	4	0.002795	0.01705622	CDKN1C, CDKN2A, CDKN1B, CDKN2C
**GOTERM_BP_DIRECT**	GO:0042632~cholesterol homeostasis	9	0.006289	0.01738668	TMEM97, APOA1, FABP4, SCARB1, CETP, MYLIP, CD24, LIPC, MTTP
**GOTERM_CC_DIRECT**	GO:0005739~mitochondrion	85	0.059393	0.01826027	MRPL40, CYB5R3, ACOX2, XRCC3, ALDH1L1, HIP1R, EHHADH, CYC1, BNIP3, GJA1, RAB1B, MMP2, MRPS31, PDHB, AKT1, ALAS1, MSRA, NDUFS4, CDKN2A, DHX34, CAT, DDAH2, CASP1, CRY1, SUCLA2, COX17, CASP2, NOL3, CRYAB, LIG1, OTC, TAZ, RAP1GDS1, CLIC1, DECR1, PNPLA4, DDIT4, MARK2, LAP3, NFU1, ALDH7A1, CAPN10, C1QBP, HARS, PCCB, PPP1R15A, SLC27A2, DEGS1, MPST, HSD17B10, PACS2, CCDC90B, PUS1, MRPS10, ADH5, SOX4, HCFC1, SFXN1, HSPA1A, SFN, ANXA6, MTCH1, MTCP1, PPP3CA, MRPS27, GCDH, SHMT2, GABARAPL1, MRPS28, C14ORF159, VHL, BCKDHB, GRSF1, MTRF1L, BCS1L, CCT7, MRPL22, FYN, PTCD3, KCNJ8, MAPK14, SDHD, MAT2B, USP48, ABL1
**GOTERM_BP_DIRECT**	GO:0031295~T cell costimulation	10	0.006987	0.01957787	AKT1, PTPN6, FYN, CCL21, LCK, RAC1, CCL19, CD4, CD24, YES1
**GOTERM_BP_DIRECT**	GO:0032355~response to estradiol	11	0.007686	0.01959793	CDKN1B, TACR3, CRYAB, EZH2, ANXA1, CD4, RARA, CAT, MMP15, IGFBP2, NCOR2
**GOTERM_MF_DIRECT**	GO:0008270~zinc ion binding	78	0.054502	0.01968289	PPARD, LMO2, RBM4, ADH1B, ZNRF4, ADH1A, APOBEC3G, RNF187, MYLIP, MMP2, MMP1, SHH, RBM4B, MBTD1, ZNF146, RGN, RBCK1, LONRF3, RARA, USP16, PHC2, ARHGEF2, ZCCHC2, RING1, MMP15, RBBP6, GTF2B, MORC2, TTF2, PJA2, RNF130, TRIM36, TRIM33, CA9, BAZ1B, TRIM34, MTF2, RNF24, ZFPM2, SIAH1, USP22, CA1, ADAMTS2, ADAMTS5, TRAF2, IFIH1, ZFAND3, RAG1, LANCL1, ADH5, FHL2, DPYS, ADH6, SP110, ZNF330, TK1, LOC440434, CYLD, TCF20, LPXN, CD4, GLO1, PEX12, RNF13, DCTD, NUP153, TRIM27, SAMHD1, WHSC1, EHMT2, TRIM22, RLF, PHF1, AIRE, PHF21A, ZNF318, RERE, BARD1
**GOTERM_BP_DIRECT**	GO:0043547~positive regulation of GTPase activity	42	0.029347	0.01974397	FGD1, FGFR3, RAP1GAP, ERBB3, HPS4, EZH2, ARHGAP15, FLCN, LLGL2, RASAL2, RCBTB2, TBC1D17, RGS12, RAC1, RAPGEF6, RGN, SHC1, BCAS3, PLCB1, SHC3, CDC42EP3, RASA1, ARHGEF10L, TBC1D3, ICAM1, GDI1, IL2RB, ARHGEF2, DENND1A, RAP1GDS1, NCKAP1L, CCL19, RACGAP1, RIC8B, CCL16, ARHGAP25, FARP2, FNBP1L, FYN, TBXA2R, SPTB, ARHGAP10
**GOTERM_CC_DIRECT**	GO:0016323~basolateral plasma membrane	17	0.011879	0.01999301	ATP1B1, AQP9, ERBB3, LEPR, FLOT1, ANXA1, SLC7A8, ATP1A1, MTTP, SLC10A1, SLC29A1, SLC26A6, CA9, OTOF, SLC2A1, SLC4A2, SLC22A1
**GOTERM_BP_DIRECT**	GO:0043552~positive regulation of phosphatidylinositol 3-kinase activity	6	0.004192	0.0207106	CCR7, FGFR3, FLT1, CCL21, RAC1, CCL19
**GOTERM_BP_DIRECT**	GO:0050853~B cell receptor signaling pathway	8	0.00559	0.02111083	PTPRC, PTPN6, LCK, NCKAP1L, IGLL1, CD79A, ABL1, PLEKHA1
**GOTERM_BP_DIRECT**	GO:0019835~cytolysis	5	0.003494	0.02139523	PRF1, C7, MICB, GZMA, MMD
**GOTERM_BP_DIRECT**	GO:0006699~bile acid biosynthetic process	5	0.003494	0.02139523	ACOX2, AKR1C4, CH25H, SLC27A2, SLC27A5

**GOTERM_BP_DIRECT**	GO:1900182~positive regulation of protein localization to nucleus	5	0.003494	0.02139523	AKT1, CDKN2A, FYN, CD2AP, GTSE1
**GOTERM_BP_DIRECT**	GO:2000378~negative regulation of reactive oxygen species metabolic process	5	0.003494	0.02139523	HIF1A, G6PD, CRYAB, HK2, BNIP3
**GOTERM_BP_DIRECT**	GO:0006069~ethanol oxidation	4	0.002795	0.02165271	ADH5, ADH6, ADH1B, ADH1A
**GOTERM_BP_DIRECT**	GO:0000050~urea cycle	4	0.002795	0.02165271	CEBPA, OTC, CPS1, SLC25A15
**GOTERM_MF_DIRECT**	GO:0008601~protein phosphatase type 2A regulator activity	5	0.003494	0.02166747	PPP2R3B, PPME1, PPP2R5D, IGBP1, ENSA
**GOTERM_CC_DIRECT**	GO:0005789~endoplasmic reticulum membrane	58	0.040527	0.02231353	CYB5R3, CYB5R4, CLSTN1, MAN1B1, PKMYT1, ALG5, GJA1, RAB1B, ZNRF4, ALG8, RTN3, SSR1, PTGIS, PLOD1, PLOD2, PIGG, CH25H, SAR1B, DDOST, AGPAT2, HPD, ATG9A, LPGAT1, PGAP1, PIGP, TMEM106C, PJA2, OTOF, ORMDL2, PPP1R15A, SLC27A2, PROS1, SLC27A5, DEGS1, FAM69A, TMCC1, CYP26B1, TMED1, RAC1, SLC39A7, CD4, ARL6IP5, RNF13, SLC39A1, FLRT3, SREBF1, FLRT2, CYP21A2, TMEM110, ITPR3, TMPRSS3, CYP4A11, CLGN, ATP2A2, ATP2A3, SPCS1, NOMO2, MGST2
**GOTERM_CC_DIRECT**	GO:0016324~apical plasma membrane	24	0.01677	0.02249683	ABCA7, ATP1B1, CLCA4, ERBB3, MPDZ, FLOT1, ANXA1, DUOX1, GJA1, ATP1A1, SLC9A3R1, PTPRO, GNAT3, SLC29A1, EPS15, CYP4A11, SLC26A6, SLC2A2, SLC2A1, RAPGEF6, SLC4A2, STC1, IGFBP2, ITPK1
**GOTERM_CC_DIRECT**	GO:0012506~vesicle membrane	5	0.003494	0.02273101	TRAF2, CLCN3, SLC26A6, ATP2A2, WAS
**GOTERM_BP_DIRECT**	GO:0001558~regulation of cell growth	10	0.006987	0.02273237	TMEM97, DDR1, KIAA1109, FAM107A, CLSTN1, IGFBP6, ESM1, IGFBP2, IGFBP3, EBAG9
**GOTERM_BP_DIRECT**	GO:0000398~mRNA splicing, via spliceosome	20	0.013975	0.02285597	RALY, CSTF3, FIP1L1, PRPF4B, U2AF2, PTBP1, TRA2A, DGCR14, SF3B4, SF3B2, UBL5, UPF3B, SNRPB, SRRM1, DHX16, SNRPA, GPATCH1, LSM2, SNRPF, HSPA8
**GOTERM_CC_DIRECT**	GO:0005887~integral component of plasma membrane	89	0.062188	0.02312833	AQP9, PCDHA2, TACR3, SLC20A1, EFNA1, TNFSF15, SLC7A8, GJA1, AQP7, CD53, TLR6, GJA4, CD48, ACVR1B, SLC1A5, HTR1B, SLC2A6, TNFRSF11A, SLC2A2, IL4R, SLC2A1, KCNK5, SLC4A2, EVI2B, HTR1F, SLC22A1, ICAM1, PTPRF, CLCA4, GPR171, RXFP3, MMP15, PTPRO, CD163, LILRB2, DDR1, SLC26A6, THBD, CCR5, CCR2, SIGLEC7, VAMP5, EFNA4, JAM2, CLEC5A, DEGS1, SLC38A4, FGFR3, ERBB3, ADORA2A, FCER2, MMD, GPR65, LANCL1, FPR1, HFE, SLCO2A1, SLC29A1, CD2, SCARB1, SLC31A2, SELPLG, GAL3ST1, CSF1R, FLRT3, MRC1, PTPRC, FLRT2, IL2RB, FLT1, SELL, AXL, CD1C, TRIM27, NCKAP1L, SLC6A15, ITPR3, EPHA3, SLC10A1, SLC16A3, P2RX5, P2RY10, ATP2A2, ATP2A3, SLC6A8, SLC6A5, TBXA2R, SLC13A1, CD79A
**GOTERM_BP_DIRECT**	GO:0016032~viral process	25	0.017469	0.02363009	CEBPA, PACS2, IL2RB, NUP153, MICB, IL16, MPDZ, NUP88, NUP188, SP110, DAXX, GTF2B, MMP1, SF3B2, RTN3, C1QBP, NUP62, HDAC1, DDX11, FYN, CCR2, LCK, RBCK1, SHC1, HSPA8
**GOTERM_MF_DIRECT**	GO:0008094~DNA-dependent ATPase activity	6	0.004192	0.02387567	XRCC3, XRCC2, DDX11, CCNH, TOP2A, TTF2
**GOTERM_BP_DIRECT**	GO:0032496~response to lipopolysaccharide	16	0.01118	0.02399506	ATP4B, ADH5, DCN, CPS1, PCK1, CCR7, THBD, TNFRSF11A, ADM, HDAC1, KCNJ8, SRR, CASP1, LOXL1, EPO, MGST2
**GOTERM_BP_DIRECT**	GO:0042541~hemoglobin biosynthetic process	3	0.002096	0.02430539	INHBA, HIF1A, EPO
**GOTERM_MF_DIRECT**	GO:0042609~CD4 receptor binding	3	0.002096	0.02448274	FYN, LCK, SPG21
**GOTERM_MF_DIRECT**	GO:0019904~protein domain specific binding	19	0.013276	0.02462117	CSNK2B, ATP1A1, SFN, ZBTB16, ARFIP1, MRPS31, NLRP1, TUBB, CEP250, NEDD4, GSN, HOMER3, IGBP1, TDG, HGS, RARA, HIST1H4C,

					KCTD13, CASP2
**GOTERM_CC_DIRECT**	GO:0043231~intracellular membrane-bounded organelle	40	0.02795	0.02476833	ACOX2, ABCA7, AQP9, HIP1R, TRA2A, FOXK2, VGF, TIPRL, LLGL2, SPG21, SLC4A1AP, SCARB1, CAT, DDOST, EXOC2, TNPO3, GTPBP2, KLF5, DTL, SNAPC1, LIG1, BYSL, ATP1A1, SLC9A3R1, MCM3, CCNL2, EPS15, CYP4A11, G6PD, NUP62, TRIM33, NCOA6, RFX1, HGS, NEU1, CLEC7A, MYBBP1A, MGST2, ATP8A1, BARD1
**GOTERM_BP_DIRECT**	GO:0006094~gluconeogenesis	7	0.004891	0.02536809	ALDOA, GPD2, ENO2, CRY1, PGK1, PCK1, ENO1
**GOTERM_BP_DIRECT**	GO:0098609~cell-cell adhesion	23	0.016071	0.025842	FLRT3, ALDOA, LDHA, PPME1, STK38, STK24, SNX2, HCFC1, HSPA1A, ARFIP1, SFN, CAPZB, MARK2, TMEM2, EPS15, TMEM47, CRKL, CSNK1D, FNBP1L, NDRG1, HSPA8, SH3GL1, ENO1
**GOTERM_MF_DIRECT**	GO:0016874~ligase activity	23	0.016071	0.02605356	TRAF2, LIG1, ZNF451, TRIM27, RAG1, CBX4, RING1, ZNRF4, RNF187, MYLIP, TRIM22, RBBP6, PJA2, RNF130, TRIM36, TRIM33, NEDD4, RBCK1, SIAH1, SUCLA2, PCCB, RNF13, BARD1
**GOTERM_BP_DIRECT**	GO:0015758~glucose transport	6	0.004192	0.02657803	AKT1, PPARD, SLC2A2, SLC2A1, HK2, YES1
**GOTERM_CC_DIRECT**	GO:0001772~immunological synapse	6	0.004192	0.0266368	ICAM1, CORO1A, SNX27, GZMA, LCK, CD53
**GOTERM_BP_DIRECT**	GO:0071285~cellular response to lithium ion	4	0.002795	0.02708158	CEBPA, CDKN1B, FABP4, SHH
**GOTERM_BP_DIRECT**	GO:0007169~transmembrane receptor protein tyrosine kinase signaling pathway	11	0.007686	0.02735221	DDR1, DOK2, FLT1, FYN, ERBB3, LCK, CD4, TNK1, YES1, SHC3, CSF1R
**GOTERM_BP_DIRECT**	GO:0030838~positive regulation of actin filament polymerization	7	0.004891	0.02802846	ICAM1, CCR7, CCL21, RAC1, NCKAP1L, LMOD1, CDC42EP3
**GOTERM_BP_DIRECT**	GO:0035924~cellular response to vascular endothelial growth factor stimulus	5	0.003494	0.02917086	AKT1, FLT1, MAPK14, VEGFA, GAS1
**GOTERM_BP_DIRECT**	GO:0030316~osteoclast differentiation	5	0.003494	0.02917086	TNFRSF11A, MAPK14, GLO1, CSF1R, FARP2
**GOTERM_BP_DIRECT**	GO:0007507~heart development	17	0.011879	0.02927915	FLRT3, PPARD, ERBB3, TAZ, SOX4, GJA1, BICC1, SRF, SHH, DVL1, CRKL, ADM, KCNJ8, NCOA6, SHC1, COX17, IFT140
**GOTERM_BP_DIRECT**	GO:0045893~positive regulation of transcription, DNA-templated	38	0.026552	0.02947753	E2F3, PPARD, ELF3, GRIP1, TFEB, FHL2, SOX4, RNF187, NFYA, ZBTB16, SHH, NPAS2, CDKN2A, BCL3, RARA, USP16, PLCB1, CREBL2, EPO, KLF5, PCBD1, VHL, PSRC1, DVL1, CDKN1C, RLF, ATF5, INHBA, MED4, HIF1A, NUP62, HDAC1, ETS2, NCOA6, AIRE, USP22, KLF2, NEK4
**GOTERM_CC_DIRECT**	GO:0030496~midbody	13	0.009084	0.02979538	TRIOBP, GDI1, CHMP4A, PSRC1, RACGAP1, FLCN, ZNF330, KIF13A, CYLD, CDCA8, DDX11, INCENP, SLC2A1
**GOTERM_BP_DIRECT**	GO:0006606~protein import into nucleus	8	0.00559	0.03003183	NUP153, NUP62, NUP88, CFL1, PTTG1IP, NUP188, PPP3CA, KPNA3
**GOTERM_CC_DIRECT**	GO:0002102~podosome	5	0.003494	0.03050669	LPXN, SH3PXD2A, GSN, LCP1, SH3GL1
**GOTERM_BP_DIRECT**	GO:0071260~cellular response to mechanical stimulus	9	0.006289	0.0305232	AKT1, RAC1, BNIP3, GJA1, ATP1A1, CASP1, CASP2, CRADD, BMP6
**GOTERM_BP_DIRECT**	GO:0071347~cellular response to interleukin-1	9	0.006289	0.0305232	ICAM1, HIF1A, PTGIS, CCL21, CAMP, CCL19, KLF2, CCL16, PCK1
**GOTERM_MF_DIRECT**	GO:0098641~cadherin binding involved in cell-cell adhesion	24	0.01677	0.03055112	ALDOA, LDHA, PPME1, STK38, STK24, SNX2, ANXA1, HCFC1, HSPA1A, ARFIP1, SFN, CLIC1, CAPZB, CD2AP, MARK2, TMEM2, EPS15, CRKL, CSNK1D, FNBP1L, NDRG1, HSPA8, SH3GL1, ENO1

**GOTERM_BP_DIRECT**	GO:0007596~blood coagulation	17	0.011879	0.03058646	ADORA2A, HPS4, F9, CAPZB, WAS, SHH, P2RX5, THBD, HDAC1, FYN, RAC1, SERPINC1, H3F3A, PHF21A, ZFPM2, ITPK1, PROS1
**GOTERM_BP_DIRECT**	GO:0006260~DNA replication	15	0.010481	0.03132115	CDC6, SSRP1, POLH, DTL, NASP, LIG1, BRIP1, RAD9A, MCM3, RBBP6, TIMELESS, NCOA6, KCTD13, REPIN1, BARD1
**GOTERM_BP_DIRECT**	GO:0048010~vascular endothelial growth factor receptor signaling pathway	9	0.006289	0.03283699	VEGFC, FLT1, FYN, MAPK13, MAPK14, VEGFA, RAC1, AXL, NCKAP1L
**GOTERM_BP_DIRECT**	GO:0051016~barbed-end actin filament capping	4	0.002795	0.03316484	TRIOBP, GSN, VIL1, CAPZB
**GOTERM_BP_DIRECT**	GO:2000147~positive regulation of cell motility	4	0.002795	0.03316484	CCR7, CCL21, CCL19, CSF1R
**GOTERM_BP_DIRECT**	GO:0031648~protein destabilization	6	0.004192	0.03340889	CDKN2A, GSN, CDC73, SIAH1, MYLIP, NOMO2
**GOTERM_BP_DIRECT**	GO:0045648~positive regulation of erythrocyte differentiation	5	0.003494	0.03360797	ACVR1B, INHBA, HIF1A, MAPK14, NCKAP1L
**GOTERM_BP_DIRECT**	GO:0030099~myeloid cell differentiation	5	0.003494	0.03360797	CDKN1C, CEBPA, NCOA6, ZBTB16, CLEC5A
**GOTERM_BP_DIRECT**	GO:0045860~positive regulation of protein kinase activity	7	0.004891	0.03388565	PTPRC, CCR7, CCL21, MMD, CCL19, CD4, DAXX
**GOTERM_BP_DIRECT**	GO:0045600~positive regulation of fat cell differentiation	7	0.004891	0.03388565	KLF5, CEBPA, AKT1, PPARD, ZBTB16, TPH1, CREBL2
**GOTERM_BP_DIRECT**	GO:0006338~chromatin remodeling	10	0.006987	0.03434945	CHD1L, HDAC1, MORF4L2, SMARCD1, TTF1, DEK, TAF6L, SCMH1, DAXX, RERE
**GOTERM_BP_DIRECT**	GO:0070126~mitochondrial translational termination	10	0.006987	0.03434945	MRPL40, MRPL2, MRPS27, MRPL22, MRPS28, MRPS18A, PTCD3, MRPS10, MTRF1L, MRPS31
**GOTERM_BP_DIRECT**	GO:0007067~mitotic nuclear division	21	0.014674	0.03484801	CDC6, TRIOBP, ARHGEF2, CCNK, NUP88, KNTC1, PKMYT1, NEDD9, CDC23, CDC20, ENSA, CEP63, CD2AP, KIF2C, TIMELESS, NUP62, INCENP, MAPRE2, NEK4, ABL1, USP16
**GOTERM_BP_DIRECT**	GO:0006406~mRNA export from nucleus	11	0.007686	0.03496558	FIP1L1, NUP153, UPF3B, NUP62, NUP88, SMG5, U2AF2, SHFM1, SRRM1, NUP188, THOC2
**GOTERM_BP_DIRECT**	GO:0045944~positive regulation of transcription from RNA polymerase II promoter	65	0.045418	0.0351061	HLF, RNASEL, ELF3, LMO2, FOXK2, MORF4L2, FLCN, SHH, AKT1, ACVR1B, CDKN2A, HEY1, POU5F1, SERPINE1, RARA, USP16, TOP2A, ARHGEF2, CCNK, CCNH, LDB2, CCNL2, HES1, INHBA, HIF1A, MTF2, IGBP1, NCOA6, VEGFA, ZFPM2, MAPK7, ZNF613, TAF9B, TFEB, SOX4, HCFC1, CDC73, DCN, SRF, NPAS2, TCF20, IL23A, MEIS2, BCL3, ETV1, PPP3CA, BCAS3, YES1, KLF5, SREBF1, CEBPA, MAFB, CEBPG, TEAD3, RLF, ATF5, GCM2, WDR61, HDAC1, ETS2, MAPK14, AIRE, KLF2, RERE, BMP6
**GOTERM_BP_DIRECT**	GO:0006360~transcription from RNA polymerase I promoter	3	0.002096	0.0352141	CEBPA, UBTF, POLR1C
**GOTERM_BP_DIRECT**	GO:0010324~membrane invagination	3	0.002096	0.0352141	FNBP1L, CHMP4A, HGS
**GOTERM_BP_DIRECT**	GO:0006929~substrate-dependent cell migration	3	0.002096	0.0352141	FMNL1, VEGFC, ANKS1A
**GOTERM_BP_DIRECT**	GO:0021930~cerebellar granule cell precursor proliferation	3	0.002096	0.0352141	ATF5, RERE, SHH
**GOTERM_CC_DIRECT**	GO:0000790~nuclear chromatin	17	0.011879	0.03523282	PPARD, POGZ, DFFA, NASP, EZH2, EHMT2, UBE2B, SRF, KLHDC3, TIMELESS, HDAC1, DDX11, POU5F1, RARA, PLCB1, ASF1B, NCOR2
**GOTERM_BP_DIRECT**	GO:0045444~fat cell differentiation	9	0.006289	0.03526736	INHBB, CEBPA, SREBF1, ATF5, RNASEL, OSBPL11, PLCB1, FBXO9, SDF4
**GOTERM_MF_DIRECT**	GO:0004872~receptor activity	19	0.013276	0.03544819	MRC1, ICAM1, IL18RAP, SLC20A1, RRBP1, MMD, TLR6, PAQR4, CD48, OGFRL1, LILRB2, SLC1A5, MED4, THBD, TNFRSF11A, SIGLEC7, CD2, CD4, TNPO3

**GOTERM_MF_DIRECT**	GO:0004024~alcohol dehydrogenase activity, zinc-dependent	3	0.002096	0.03546629	ADH6, ADH1B, ADH1A
**GOTERM_BP_DIRECT**	GO:0048469~cell maturation	6	0.004192	0.03719681	HES1, CEBPA, CCL21, VEGFA, AXL, CCL19
**GOTERM_MF_DIRECT**	GO:0019901~protein kinase binding	29	0.020264	0.03761202	TRAF2, PPME1, TRIB3, SFN, DAXX, CYLD, CDKN2A, CEP250, CDKN2C, RAC1, CD4, CD24, CRY1, EXOC2, SREBF1, PTPN6, PTPRC, CCNK, RAD9A, ATP1A1, RACGAP1, TRIM22, WAS, CAMK2N1, CCNL2, DVL1, HIF1A, LCK, PPP1R15A
**GOTERM_CC_DIRECT**	GO:0005913~cell-cell adherens junction	25	0.017469	0.03776838	ALDOA, LDHA, PPME1, STK38, SNX2, HCFC1, HSPA1A, SFN, CD2AP, CAPZB, NDRG1, HSPA8, ENO1, STK24, FLOT1, ANXA1, ARFIP1, CLIC1, TMEM2, MARK2, EPS15, CRKL, CSNK1D, FNBP1L, SH3GL1
**GOTERM_BP_DIRECT**	GO:0019433~triglyceride catabolic process	5	0.003494	0.03841615	APOA1, FABP4, PNPLA4, CPS1, LIPC
**GOTERM_CC_DIRECT**	GO:0005694~chromosome	11	0.007686	0.03909356	THAP7, SSRP1, PRPF4B, HMGB3, MDC1, DTL, INTS7, WHSC1, EHMT2, LOXL2, RBBP6
**GOTERM_CC_DIRECT**	GO:0005730~nucleolus	56	0.03913	0.03946703	MRPL40, MORF4L2, RBM4, DAXX, CD2AP, MRPS31, RBM4B, CRYL1, CDCA8, CDKN2A, DDX11, ZNF146, TOP2A, NOL3, SNAPC1, DTL, STK24, BYSL, PTBP1, TTF1, RBBP6, GTF2B, RCL1, TIMELESS, CA9, C1QBP, ABT1, CSTB, SNRPA, MYBBP1A, C1D, REV3L, TRA2A, MKNK2, TRIB3, SP110, ZBTB16, ZNF330, TSPYL1, BCAS3, HSPA8, TSEN34, CEBPA, SSRP1, PTPN6, NUP153, MKI67, PAK1IP1, CPS1, ITPR3, TULP3, CRBN, UPF3B, UBTF, PPID, ABL1
**GOTERM_BP_DIRECT**	GO:0006954~inflammatory response	29	0.020264	0.03978671	ELF3, ADORA2A, LY86, FPR1, TLR6, CXCL12, AKT1, TNFRSF11A, IL23A, FOLR2, CCL21, CYP26B1, RAC1, CSF1R, IL18RAP, LIPA, ANXA1, AXL, CCL19, CD5L, CCL16, NLRP1, SIGLEC1, CCR7, CCR5, CCR2, TBXA2R, CLEC7A, BMP6
**GOTERM_BP_DIRECT**	GO:0034375~high-density lipoprotein particle remodeling	4	0.002795	0.03989397	APOA1, SCARB1, CETP, LIPC
**GOTERM_BP_DIRECT**	GO:0050930~induction of positive chemotaxis	4	0.002795	0.03989397	VEGFC, IL16, VEGFA, CXCL12
**GOTERM_BP_DIRECT**	GO:0007623~circadian rhythm	9	0.006289	0.04048613	SREBF1, ATF5, RBM4B, NPAS2, TIMELESS, HS3ST2, SERPINE1, TPH1, MTTP
**GOTERM_BP_DIRECT**	GO:0000902~cell morphogenesis	8	0.00559	0.04119318	ARHGEF2, LST1, LIPA, VHL, MAPK14, TBCC, KLF2, PTPRO
**GOTERM_CC_DIRECT**	GO:0005615~extracellular space	83	0.057996	0.04119367	IL16, KERA, LY86, IGFBP6, TNFSF15, LRRC17, VGF, MMP2, CXCL12, SHH, RTN3, TTR, APOA1, PTGIS, SEMA3G, GSN, IL4R, SERPINE1, CETP, CAT, VWA1, LOXL2, ANGPT2, LOXL1, ICAM1, F12, ACTN4, CAMP, F9, PDE4C, CLIC1, PRELP, INHBB, VEGFC, LILRB2, DDR1, THBD, C1QBP, ADM, INHBE, CTSO, CFL1, VEGFA, CSTB, STC1, TNFAIP2, PROS1, LCP1, ADAMTS5, ALDOA, ERBB3, HDGF, HFE, DCN, SFN, CCL27, CCL21, ENO2, SERPINC1, C2, LECT2, HSPA8, DPT, EPO, ENO1, FLRT3, FLRT2, FLT1, EFEMP1, AXL, ANXA1, CCL19, CD5L, CCL16, KRT35, OMD, CLEC3B, LIPC, IGFBP2, GDF15, IGFBP3, BMP6, DMBT1
**GOTERM_BP_DIRECT**	GO:0002576~platelet degranulation	11	0.007686	0.04156579	ALDOA, VEGFC, APOA1, PLEK, ACTN4, CLEC3B, VEGFA, SERPINE1, PROS1, CDC37L1, SPP2
**GOTERM_MF_DIRECT**	GO:0051015~actin filament binding	13	0.009084	0.04324063	FMNL1, TRIOBP, ACTN4, HIP1R, VIL1, CAPZB, SYNE1, CORO1A, CFL1, WIPF2, ABL1, LCP1, SPTB
**GOTERM_BP_DIRECT**	GO:0030324~lung development	9	0.006289	0.0432783	HES1, CEBPA, SREBF1, LIPA, TIMELESS, VEGFA, ZFPM2, ADAMTS2, SHH
**GOTERM_BP_DIRECT**	GO:0051301~cell division	27	0.018866	0.04353138	KIFC1, TRIOBP, KNTC1, NEDD9, CDC73, ENSA, CD2AP, LLGL2, KIF2C, CDCA8, TUBB, NCAPG, KLHL22, USP16, CDC6, CCNK, ARHGEF2, POGZ, LIG1, PSRC1, CDC23, CDC20, CEP63, TACC3, TIMELESS, MAPRE2, NEK4
**GOTERM_BP_DIRECT**	GO:0051491~positive regulation of filopodium assembly	5	0.003494	0.04359681	CCR7, FNBP1L, CCL21, BCAS3, SRF

**GOTERM_BP_DIRECT**	GO:0030041~actin filament polymerization	5	0.003494	0.04359681	GSN, VIL1, RAC1, ABL1, WAS
**GOTERM_CC_DIRECT**	GO:0035098~ESC/E(Z) complex	4	0.002795	0.04379141	PHF1, JARID2, MTF2, EZH2
**GOTERM_BP_DIRECT**	GO:0045814~negative regulation of gene expression, epigenetic	7	0.004891	0.04406128	PHF1, JARID2, MTF2, EZH2, TRIM27, H3F3A, HIST1H4C
**GOTERM_MF_DIRECT**	GO:0046875~ephrin receptor binding	5	0.003494	0.04411751	ANKS1A, FYN, EFNA1, SHC1, EFNA4
**GOTERM_CC_DIRECT**	GO:0015630~microtubule cytoskeleton	13	0.009084	0.04422338	AKT1, KIF2C, SHMT2, MAST2, TIMELESS, CRYAB, RUSC1, KIFAP3, PSRC1, MAPRE2, NDRG1, MID1IP1, TACC3
**GOTERM_CC_DIRECT**	GO:0031519~PcG protein complex	5	0.003494	0.04477217	RING1, CSNK2B, CBX4, UBAP2L, PHC2
**GOTERM_MF_DIRECT**	GO:0004713~protein tyrosine kinase activity	13	0.009084	0.04537694	FGFR3, FLT1, ERBB3, STAT5A, AXL, BAZ1B, FYN, CLK2, MAP3K9, LCK, TNK1, ABL1, YES1
**GOTERM_BP_DIRECT**	GO:0042517~positive regulation of tyrosine phosphorylation of Stat3 protein	6	0.004192	0.0455332	HES1, FGFR3, IL23A, HDAC1, VEGFA, CSF1R
**GOTERM_MF_DIRECT**	GO:0004714~transmembrane receptor protein tyrosine kinase activity	6	0.004192	0.04616505	DDR1, FLT1, ERBB3, TRIM27, AXL, CSF1R
**GOTERM_BP_DIRECT**	GO:0006397~mRNA processing	16	0.01118	0.0466179	KHDRBS1, RNASEL, U2AF2, RBM4, PTBP1, RBBP6, SF3B4, TTF2, SF3B2, RBM4B, C1QBP, SFPQ, SRRM1, GPATCH1, LSM1, TSEN34
**GOTERM_BP_DIRECT**	GO:0008209~androgen metabolic process	4	0.002795	0.04725571	AKR1C4, ADM, SHH, PLEKHA1
**GOTERM_BP_DIRECT**	GO:0030949~positive regulation of vascular endothelial growth factor receptor signaling pathway	4	0.002795	0.04725571	GRB10, HIF1A, FLT1, VEGFA
**GOTERM_CC_DIRECT**	GO:0005681~spliceosomal complex	10	0.006987	0.04738458	U2AF2, SNRPB, SNRPA, DHX16, IVNS1ABP, SNRPF, SF3B4, TTF2, HSPA8, SF3B2
**GOTERM_BP_DIRECT**	GO:0002606~positive regulation of dendritic cell antigen processing and presentation	3	0.002096	0.04762532	CCR7, CCL21, CCL19
**GOTERM_BP_DIRECT**	GO:0032515~negative regulation of phosphoprotein phosphatase activity	3	0.002096	0.04762532	PPP1R11, RGN, PPP1R15A
**GOTERM_BP_DIRECT**	GO:0043567~regulation of insulin-like growth factor receptor signaling pathway	3	0.002096	0.04762532	IGFBP6, IGFBP2, IGFBP3
**GOTERM_BP_DIRECT**	GO:0051014~actin filament severing	3	0.002096	0.04762532	FMNL1, GSN, VIL1
**GOTERM_BP_DIRECT**	GO:0030856~regulation of epithelial cell differentiation	3	0.002096	0.04762532	KIAA1109, ALOX15B, CD24
**GOTERM_MF_DIRECT**	GO:0004022~alcohol dehydrogenase (NAD) activity	3	0.002096	0.04796	ADH5, ADH6, ADH1A
**GOTERM_CC_DIRECT**	GO:0000922~spindle pole	11	0.007686	0.04843568	CDC6, NUP62, DDX11, MAPK14, PSRC1, KNTC1, NEDD9, GPSM2, CDC20, CEP63, TACC3
**GOTERM_MF_DIRECT**	GO:0001047~core promoter binding	8	0.00559	0.04848689	E2F3, NPAS2, HDAC1, SFPQ, EZH2, CRY1, MYBBP1A, GTF2B
**GOTERM_BP_DIRECT**	GO:0007568~aging	15	0.010481	0.04890511	SREBF1, TACR3, CRYAB, DCN, PCK1, AKT1, CDKN1C, ADM, SRR, RGN, CAT, IGFBP2, LOXL2, CASP2, EPO
**GOTERM_BP_DIRECT**	GO:0009653~anatomical structure morphogenesis	10	0.006987	0.04951861	MRPL40, IER3, LST1, POU5F1, LIG1, RAC1, SIAH1, WHSC1, SCMH1, KRT35

**GOTERM_MF_DIRECT**	GO:0046966~thyroid hormone receptor binding	5	0.003494	0.04972913	TAF11, MED4, NUP62, NCOA6, GTF2B
**GOTERM_MF_DIRECT**	GO:0051879~Hsp90 protein binding	5	0.003494	0.04972913	NPAS2, HIF1A, NUP62, NASP, PPID
**KEGG_PATHWAY**	hsa00010:Glycolysis / Gluconeogenesis	14	0.009782	2.42E-04	ALDOA, LDHA, PFKL, HK2, ADH5, ADH6, ADH1B, ADH1A, PDHB, PCK1, ALDH7A1, ENO2, PGK1, ENO1
**KEGG_PATHWAY**	hsa04066:HIF-1 signaling pathway	17	0.011879	3.08E-04	FLT1, PFKFB3, VHL, MKNK2, HK2, EGLN3, PDHB, AKT1, HIF1A, CDKN1B, SERPINE1, VEGFA, SLC2A1, ENO2, ANGPT2, EPO, ENO1
**KEGG_PATHWAY**	hsa01200:Carbon metabolism	18	0.012577	6.94E-04	ALDOA, SHMT2, PFKL, EHHADH, HK2, ADH5, CPS1, PDHB, G6PD, SDHD, ENO2, RGN, CAT, PGK1, SUCLA2, PCCB, PRPS2, ENO1
**KEGG_PATHWAY**	hsa05230:Central carbon metabolism in cancer	11	0.007686	0.00650896	SLC16A3, AKT1, SLC1A5, HIF1A, FGFR3, G6PD, PFKL, SLC2A2, SLC2A1, HK2, PDHB
**KEGG_PATHWAY**	hsa01130:Biosynthesis of antibiotics	24	0.01677	0.00770224	ALDOA, GCDH, HSD17B10, SHMT2, LDHA, PFKL, EHHADH, OTC, BCKDHB, HK2, ADH5, PDHB, PCK1, ALDH7A1, G6PD, SDHD, ENO2, RGN, CAT, PGK1, SUCLA2, PCCB, PRPS2, ENO1
**KEGG_PATHWAY**	hsa03320:PPAR signaling pathway	10	0.006987	0.02454067	ACOX2, PPARD, APOA1, EHHADH, FABP4, AQP7, SLC27A2, SLC27A5, MMP1, PCK1
**KEGG_PATHWAY**	hsa04110:Cell cycle	15	0.010481	0.02464567	CDC6, E2F3, CCNH, PKMYT1, CDC23, CDC20, ESPL1, SFN, MCM3, CDKN1C, CDKN1B, CDKN2A, HDAC1, CDKN2C, ABL1
**KEGG_PATHWAY**	hsa04976:Bile secretion	10	0.006987	0.0291234	ATP1B1, AQP9, ADCY7, SLC2A1, SLC4A2, SCARB1, ATP1A1, SLC27A5, SLC22A1, SLC10A1
**KEGG_PATHWAY**	hsa04520:Adherens junction	10	0.006987	0.03427458	PTPN6, RAC2, PTPRF, ACTN4, FYN, RAC1, CSNK2B, YES1, WAS, FARP2
**KEGG_PATHWAY**	hsa01230:Biosynthesis of amino acids	10	0.006987	0.03707214	ALDOA, SHMT2, PFKL, OTC, ENO2, MAT2B, CPS1, PGK1, PRPS2, ENO1
**KEGG_PATHWAY**	hsa05220:Chronic myeloid leukemia	10	0.006987	0.03707214	AKT1, E2F3, CDKN2A, CDKN1B, CRKL, HDAC1, STAT5A, SHC1, ABL1, SHC3
**KEGG_PATHWAY**	hsa00620:Pyruvate metabolism	7	0.004891	0.03842471	LDHA, ALDH7A1, ACYP1, GLO1, GRHPR, PDHB, PCK1
**KEGG_PATHWAY**	hsa04015:Rap1 signaling pathway	21	0.014674	0.04343441	FLT1, FGFR3, ADCY7, RAP1GAP, EFNA1, ADORA2A, FPR1, FARP2, AKT1, VEGFC, CRKL, RAC2, MAPK13, MAPK14, RAC1, VEGFA, RAPGEF6, EFNA4, PLCB1, ANGPT2, CSF1R
**KEGG_PATHWAY**	hsa00310:Lysine degradation	8	0.00559	0.04426413	GCDH, ALDH7A1, PLOD1, PLOD2, EHHADH, WHSC1, EHMT2, PIPOX
**KEGG_PATHWAY**	hsa00071:Fatty acid degradation	7	0.004891	0.04727144	GCDH, ALDH7A1, EHHADH, ADH5, ADH6, ADH1B, ADH1A

Notes: GO, Gene ontology; KEGG, Kyoto Encyclopedia of Genes and Genomes; BP, biological process; CC, cell component; MF, molecular function.

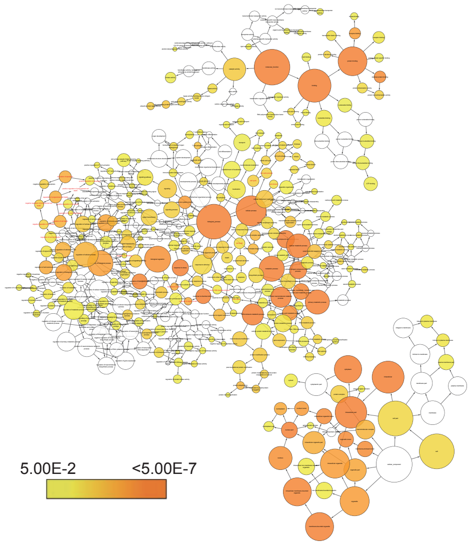

Figure S1. BinGO enrichment results of VEGF and its co-expression genes
